# Enhanced Catalytic Dechlorination of Polyvinyl Chloride (PVC) and H_2_ Production Enabled by Synergistic Ga^δ+^/Ga^0^ Active Sites in Liquid Metal Particles

**DOI:** 10.1021/jacs.6c09418

**Published:** 2026-09-02

**Authors:** Benjamin H. Crockett Zingg, Nabihan B. Abdul Rahman, Nishu Devi, Anya Zornes, Joshua T. Damron, Ernesto C. Zuleta S., Yan-Ru Lin, José David Arregui-Mena, Chang Liu, Murillo Longo Martins, Shukai Yao, Mo Li, De-en Jiang, Harry M. Meyer, Zili Wu, Felipe Polo-Garzon

**Affiliations:** † Department of Chemistry, Kentucky State University. Frankfort, Kentucky 40601, United States; ‡ Chemical Sciences Division, 6146 Oak Ridge National Laboratory, Oak Ridge, Tennessee 37831, United States; § Materials Science and Technology Division, Oak Ridge National Laboratory, Oak Ridge, Tennessee 37831, United States; ∥ Neutron Scattering Division, Oak Ridge National Laboratory, Oak Ridge, Tennessee 37831, United States; ⊥ Chemical and Biomolecular Engineering Department, 5718 Vanderbilt University, Nashville, Tennessee 37235, United States

## Abstract

Polyvinyl chloride (PVC) is ubiquitous yet challenging to recycle due to its tendency to thermally decompose above 250 °C, releasing toxic, corrosive chlorinated compounds, and its inability to melt. Here, we report a catalytic strategy for PVC upcycling at 160 °C using gallium liquid metal particles (Ga-LMP) featuring a dynamic Ga-GaOOH core–shell architecture. These catalysts enable concurrent dechlorination and hydrogen evolution, yielding up to 7% H_2_ (based on initial hydrogen atoms in PVC) along with a highly dechlorinated (>95%) carbonaceous solid and aqueous HCl. Mechanistic investigations combining X-ray photoelectron spectroscopy, infrared spectroscopy, solid-state NMR, inelastic neutron scattering, and ab initio molecular dynamics reveal a synergistic interplay between Ga^δ+^ sites in the GaOOH shell and metallic Ga^0^ in the core. Cationic Ga initiates C–Cl bond activation and HCl formation, while progressive reduction of the shell exposes Ga^0^ sites that promote C–H activation and H_2_ evolution. Control experiments with a Ga salt and bulk Ga liquid metal confirmed that neither oxidation state alone can achieve both transformations efficiently. This work establishes a dynamic dual-site paradigm for liquid metal catalysis, in which the in situ evolution and coexistence of oxidized and metallic species enable sequential and cooperative bond activation pathways. These findings provide a general design principle for novel liquid metal catalysts that target challenging polymer transformations under mild conditions.

## Graphical abstract

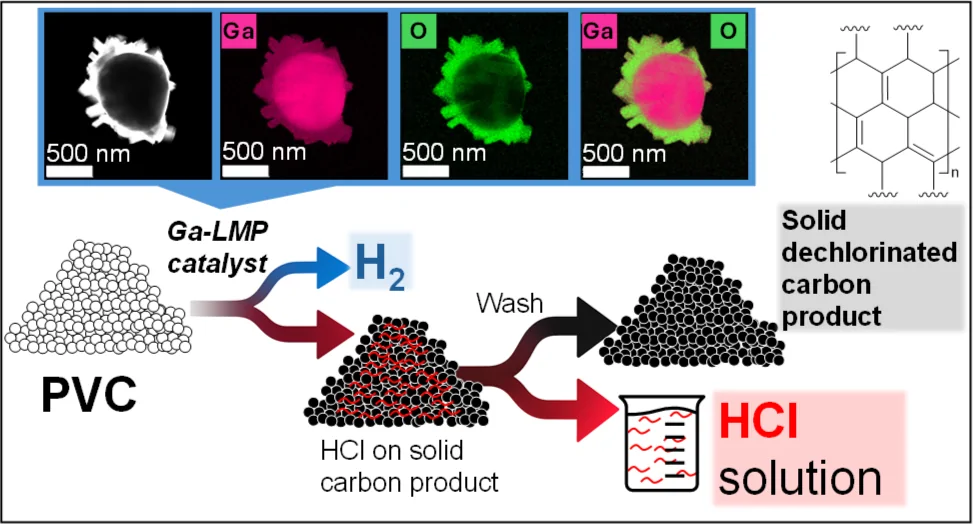

## Introduction

Plastics are an integral part of our daily lives because of their versatility and durability. However, when discarded, they represent a significant loss of valuable materials and energy. Polyvinyl chloride (PVC), in particular, is widely used in construction materials, household products, medical supplies, automotive components, and packaging, accounting for roughly 9% of global plastic production.^1–3^ For decades, researchers have studied PVC’s thermal degradation. At temperatures above 250 °C and depending on the atmosphere (i.e., H_2_, O_2_, steam, inert gas), PVC can decompose into HCl, polyenes, char, tar, low molecular weight organics, syngas, and chlorinated organic compounds with unpredictable chlorine content and molecular weight—byproducts that are hazardous to human health and rarely recycled. Overall, thermal processes suffer from poor product selectivity and high energy demands, limiting their commercial viability.^3–9^ These limitations not only complicate recycling but also restrict the integration of PVC into mixed plastic waste streams.

Catalytic chemical upcycling of PVC provides a promising alternative by enabling controlled chemical conversion at lower temperatures, thereby allowing for the targeted production of high-value chemicals. Significant progress has been made in this area; for example, PVC has been transformed into long-chain hydrocarbons with chlorine sequestered as a salt using a Pt/C catalyst under pressurized H_2_;^10^ ZnO has been employed to create porous carbon structures;^11,12^ solid oxide catalysts coupled with a tetrahydrofuran (THF)–H_2_O/decane solution have produced benzene and graphitic carbon;^13^ Ru/Al_2_O_3_ catalysts in a THF solution under H_2_ pressure have generated organic chlorides and polyethylene-like molecules;^14^ and chloroaluminate ionic liquids have been used to catalyze the coconversion of PVC and light isoalkanes to produce chlorine-free fuel range hydrocarbons and hydrogen chloride.^15^ Other studies have explored the coconversion of PVC with polymers such as polyethylene terephthalate (PET) or polypropylene using various catalytic systems, though these methods often leave behind partially dechlorinated byproducts or require difficult solid–solid separations, high-pressure hydrogen, or the extensive use of solvents.^16–18^

Addressing this challenge requires catalytic strategies capable of selectively activating strong C–Cl and C–H bonds under mild conditions, while avoiding the formation of hazardous byproducts. More broadly, this problem exemplifies a central challenge in chemical catalysis: how to design systems that orchestrate multiple, distinct bond activation steps within a single material in a controlled and cooperative manner. Developing such catalysts would enable not only efficient PVC upcycling but also a wider class of transformations involving halogenated and otherwise recalcitrant polymers. An elegant solution is offered by liquid metal (LM) catalysts, which consist of metals that melt near room temperature. These catalysts promote intimate interactions between solid reactants, such as nonmelting PVC, and catalytic sites. Recently, LM catalysts have received significant attention due to their resistance to deactivation through coking, thanks to the natural phase separation between the LM and coke, and their superior catalytic properties. Their applications range from alkane dehydrogenation^19–21^ and NaCl electrolysis (in the industrial Chlor-alkali process)^22,23^ to methane reforming,^24,25^ coal liquefaction,^26^ propylene production from long-chain hydrocarbons,^27^ methane pyrolysis to yield solid carbon and H_2_,^28–33^ and CO_2_ conversion to solid carbon.^34,35^ Building on these insights, our team has extended the use of LM catalysts to the upcycling of PVC.^36^ We demonstrated that PVC can be converted at a relatively low temperature of 200 °C, well below the uncontrolled thermal decomposition threshold of 250 °C,^5^ into H_2_, a polycyclic carbon material, and liquid HCl, thereby avoiding the corrosive effects of gaseous HCl. Importantly, this process was truly catalytic, as evidenced by the catalyst’s reusability.^36^

To unlock the full potential of LM catalysts in PVC upcycling, it is crucial to understand the active sites involved. In this work, we identify the synergistic role of Ga^δ+^/Ga^0^ sites in driving PVC dechlorination and H_2_ production. Leveraging this knowledge, we have designed Ga liquid metal particles (Ga-LMP) with a core–shell structure, consisting of a metallic Ga core and a GaOOH shell. This tailored architecture strikes the optimal balance between oxidized and metallic Ga, and its upcycling performance surpasses that of conventional bulk Ga LM. This study not only advances our approach to PVC upcycling but also exemplifies how fundamental insights into catalyst design can pave the way for enhanced polymer upcycling strategies.

## Results and Discussion

### Ga-LMP Surpasses the Performance of Bulk Ga LM Beads

PVC was converted in a batch reactor under an argon atmosphere at 160 and 200 °C, using bulk Ga LM beads or Ga-LMP. No coreactants or solvents were used during the reaction. Ga-LMP-1 h are liquid metal particles with a core–shell structure of Ga-GaOOH, synthesized under sonication for 1 h, as elaborated later in the article. After the reaction concluded, the solid product was washed with water or acetone at ambient temperature to remove HCl weakly adsorbed on the dechlorinated solid product. The reaction thus yielded three product phases: gas phase products, a solid dechlorinated polymeric product, and a chlorinated liquid product (Figure 1a). The extent of dechlorination was measured using thermogravimetric analysis coupled with mass spectrometry (TGA-MS). The gas overhead was analyzed using mass spectrometry (MS).

**Figure 1:**
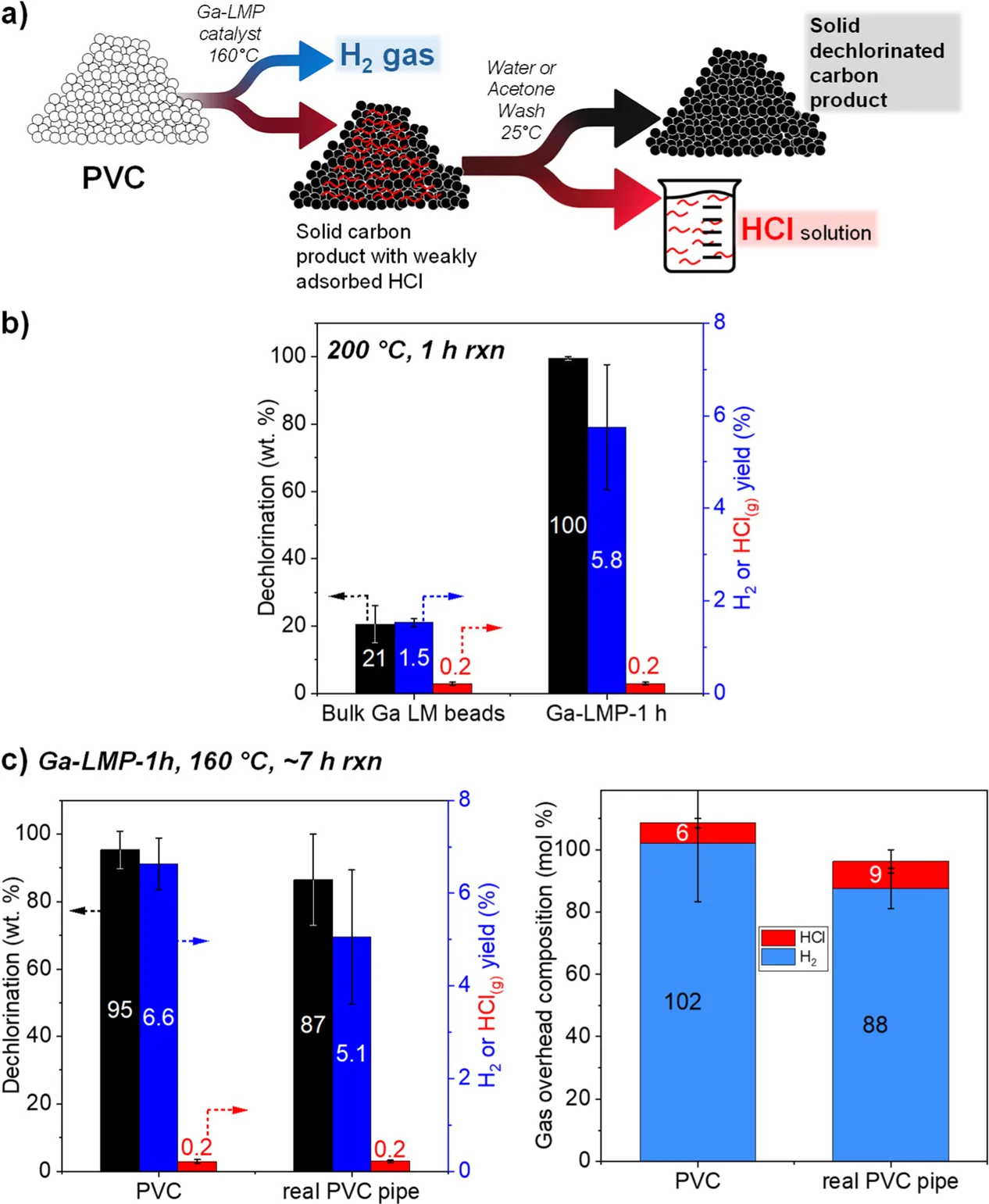
The Ga-LMP-1 h catalyst surpasses the performance of bulk Ga LM beads: greater dechlorination and H_2_ yield in the gas product overhead were attained. (a) Schematic of PVC conversion into H_2_, a solid dechlorinated carbon product, and a liquid HCl solution (water or acetone). (b) Dechlorination, H_2_, and HCl_(g)_ yield after 1 h of the reaction, using PVC (Sigma-Aldrich) and either bulk Ga LM beads or Ga-LMP-1 h as catalysts. Reaction conditions: 1 h, 200 °C, 0.2 g PVC, 0.049 g of the catalyst. (c) Dechlorination, H_2_ and HCl_(g)_ yield, and composition of the gas overhead after 6.75 ± 0.75 h of the reaction, using PVC (Sigma-Aldrich) or flakes of a discarded real PVC pipe and Ga-LMP-1 h as the catalyst. Reaction conditions: 6–7.5 h, 160 °C, 0.2 g PVC, 0.049 g Ga-LMP-1 h.

At 200 °C, Ga-LMP-1 h showed superior performance compared to bulk Ga LM beads. A 5-fold increase in dechlorination (wt %) and a 4-fold increase in H_2_ yield were obtained while suppressing undesired production of corrosive gas phase HCl. The yield of H_2_ or HCl is defined as the percentage of H atoms from initial PVC that ended up in the gas product (Figures 1b and S1). At 160 °C, with a longer reaction time (∼7 h), Ga-LMP-1h was able to attain near-full dechlorination of PVC (95%) while achieving 6.6% yield of H_2_ and limiting production of gas phase HCl. A similar performance was observed when a discarded piece of PVC pipe was upcycled, highlighting the robustness of the catalyst to the additives encountered in commercial PVC. The solid products analyzed in Figure 1b,c were obtained after washing with water or acetone the dechlorinated solid obtained after the reaction. A water wash generates a valuable aqueous HCl solution, as confirmed by the absence of any other liquid products via ^1^H and ^13^C nuclear magnetic resonance (NMR) spectroscopy (Figure S2). HCl was identified via the silver nitrate test (Figure S3a) and quantified using an aqueous NaOH solution and phenolphthalein as the indicator. The amount of Cl^–^ titrated in the water wash corresponded to the Cl removed from PVC (Figure S3b). If acetone was used for the wash instead of water, a solution of HCl, mesityl oxide, and diacetone alcohol in acetone was produced since HCl catalyzes self-condensation of acetone to diacetone alcohol, followed by dehydration to mesityl oxide (Figure S4). These products find applications as chemical intermediates, or, in the case of diacetone alcohol, as a solvent itself.^37^ The upcycling of PVC into three valuable products (gas H_2_, dechlorinated carbonaceous structure, and HCl, along with mesityl oxide and diacetone alcohol as an option) without the use of coreactants or solvents at 160 °C was attained due to the synergistic role of Ga^δ+^/Ga^0^ active sites in Ga-LMP catalysts, as illustrated below.

### The Role of Ga^δ+^/Ga^0^ Active Sites

In our previous work,^36^ PVC upcycling using bulk Ga LM was attained. The process required 200 °C and excess Ga LM to attain 90% dechlorination of PVC. Practical implementation of LM catalysts for plastics upcycling requires using the minimal amount of the catalyst necessary. To explore the minimal use of bulk Ga LM for PVC upcycling, the reaction was conducted at temperatures of 150 and 200 °C while varying the PVC concentration in the mixture from 5 wt % (mixtures dominated by Ga) up to 100 wt % (no catalyst present). To control for variations in the total mixture volume—since drastic changes in the PVC/Ga ratio could alter mass transfer conditions—the reactor was loaded with either 0.1, 0.2, or 0.3 g of PVC (Figure 2). At 200 °C, dechlorination of PVC was nearly complete over a wide range of PVC loadings; however, as the PVC concentration approached 100 wt %, the dechlorination efficiency declined. To enable a more detailed comparison of dechlorination extents across different PVC concentrations, the reaction temperature was lowered to 150 °C, resulting in partial dechlorination. Under these conditions, dechlorination levels varied broadly from negligible dechlorination to approximately 60%.

**Figure 2:**
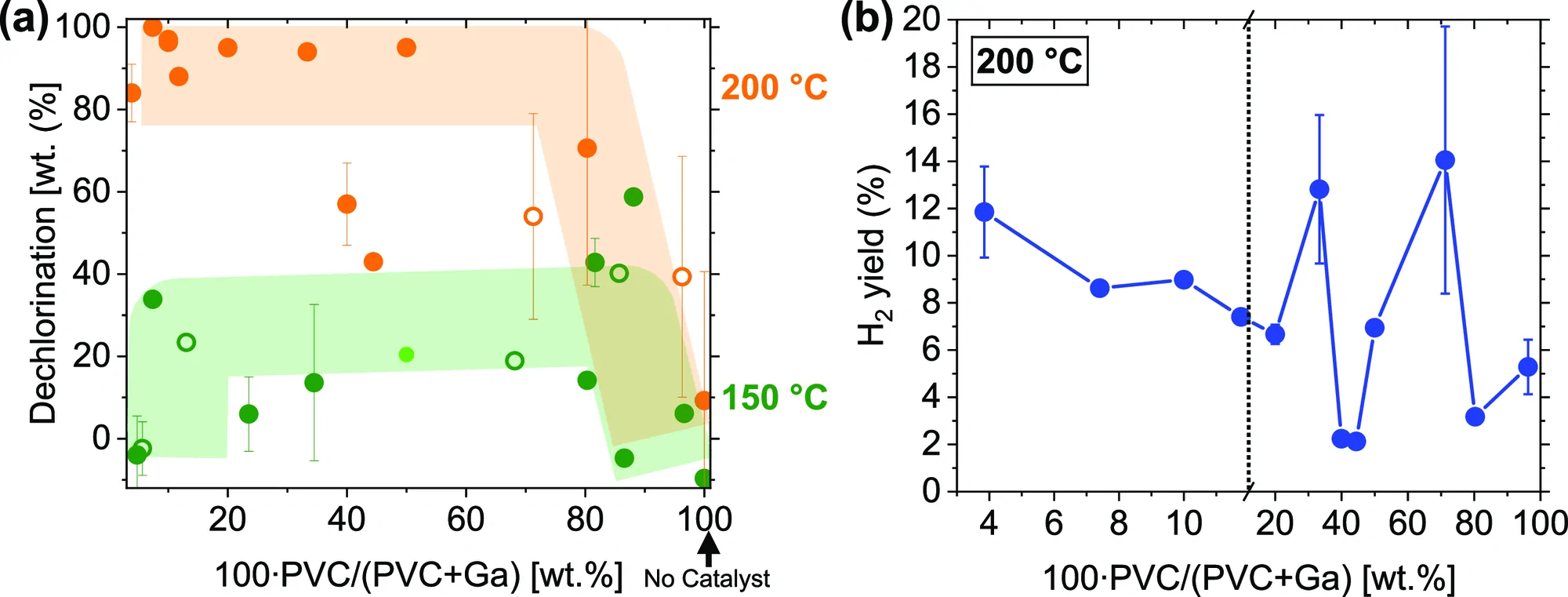
Significant uncertainties are observed in the dechlorination of PVC and yield of H_2_ at various concentrations of PVC. (a) Extent of dechlorination of the carbon product after PVC conversion at 150 and 200 °C. Orange symbols are results at 200 °C (solid orange = 0.2 g PVC, hollow orange = 0.4 g PVC). Green symbols are results at 150 °C (light solid green = 0.1 g PVC, dark solid green = 0.2 g PVC, hollow green = 0.3 g PVC). (b) H_2_ yield at 200 °C (connecting lines are meant to guide the reader). Reaction conditions: Ga LM catalyst, 0.2–0.4 g PVC, 1 h.

Despite rigorous efforts to minimize uncertainties, considerable dispersion in the extent of dechlorination, H_2_ yields, and the composition of the overhead gas (see Figures 2 and S5) persisted. The only variable that remained uncontrolled during these catalytic runs was the size of the Ga pieces introduced into the reactor (i.e., Ga beads and thin flakes). Ga readily oxidizes upon exposure to ambient air, and smaller Ga fragments present a larger interfacial area, which suggests that variations in the ratio of cationic to metallic Ga could contribute to the observed uncertainties in the catalytic performance. Ga LM has a high surface tension (700 mN m^–1^, 10 times that of water);^38^ however, a GaO_*x*_ layer could reduce surface tension and facilitate reactive wetting of PVC, where GaO_*x*_ reacts with HCl to produce GaOH_*x*_Cl_*y*_ functionalities.^39,40^ Thus, the coexistence of metallic and oxidized Ga was hypothesized to explain the uncertainties in the catalytic performance.

To test this hypothesis, we first compared the catalytic performance of bulk Ga LM beads and Ga nitrate (Ga­(NO_3_)_3_•9H_2_O) for PVC conversion (Figure 3a), where the electrostatic interaction between the chloride groups in PVC and Ga^δ+^ is expected to promote dispersion of the metal cation.^41^ A similar strategy was implemented by Bai et al.,^42^ who impregnated Zn­(CH_3_CO_3_)_2_ on polyethylene terephthalate (PET) to deconstruct the polymer via methanolysis. Although Ga nitrate is hardly recoverable after the reaction, its ability to dechlorinate PVC provides insights into the role of cationic Ga during LM-catalyzed upcycling.

**Figure 3:**
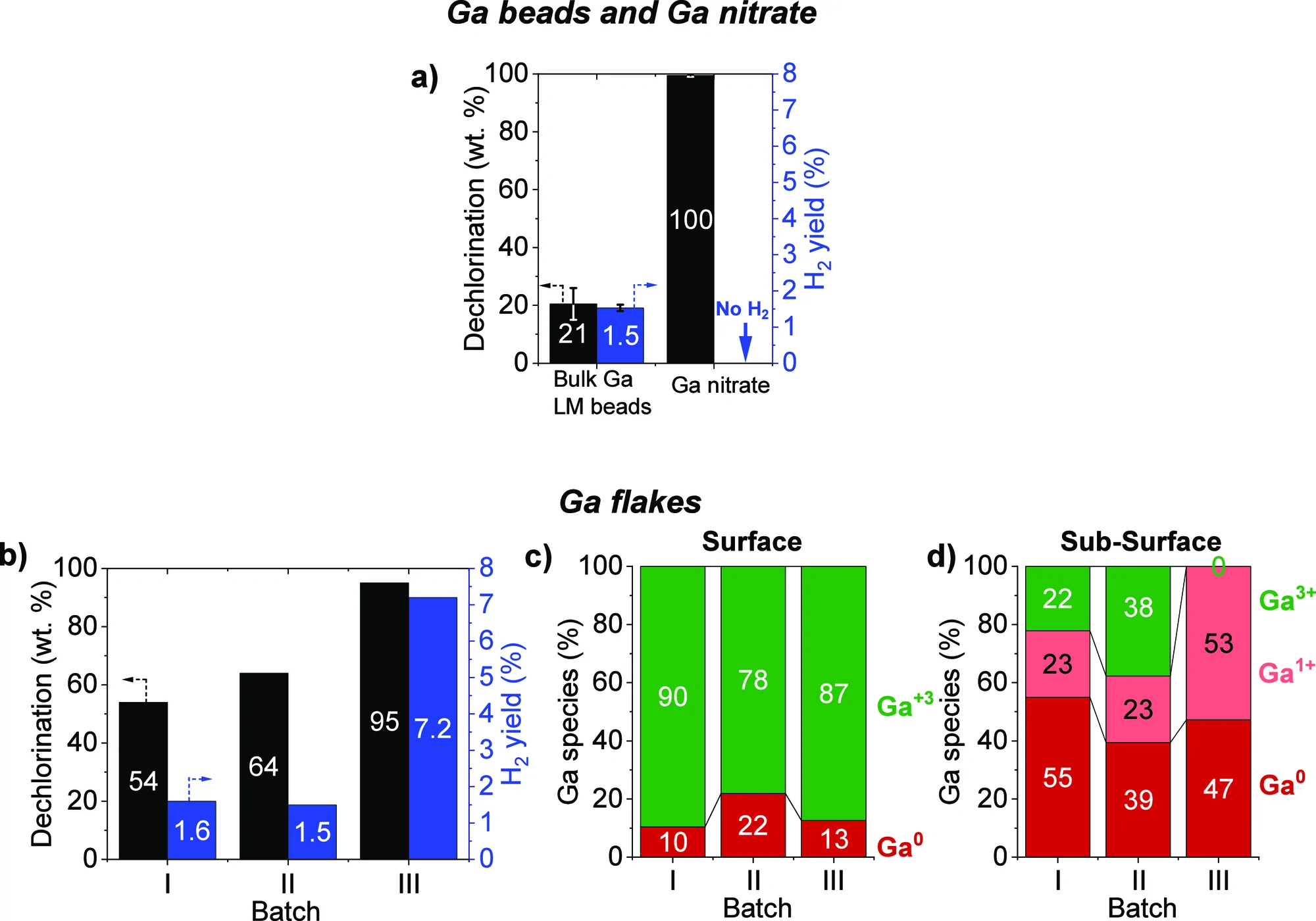
PVC conversion using bulk Ga LM beads, Ga nitrate, or Ga LM flakes. Metallic Ga was indispensable to produce H_2_. Oxidized Ga and metallic Ga worked synergistically to boost both H_2_ production and dechlorination. (a) PVC dechlorination and H_2_ yield obtained using bulk Ga LM beads and Ga nitrate (Ga­(NO_3_)_3_•9H_2_O). (b) PVC dechlorination and H_2_ yield obtained using three different batches of Ga flakes. All batches were prepared in the same fashion, except for the final storage time in a refrigerator, which was 48, 2, and 24 h for Batch I, II, and III respectively. (c,d) Ga 2p3/2 XPS analysis of Ga LM flakes on the surface (c) and subsurface (d) (after etching ∼7–8 nm of the outer surface). Binding energies: 1115.9–1116.5 eV (Ga^0^), 1117.0–1117.5 eV (Ga^1+^), 1118.1–1118.3 eV (Ga^3+^).^43–45^ Reaction conditions: 200 °C, 1 h, 0.2 g PVC, 0.049 g Ga (as Ga LM or as 0.281 g of Ga­(NO_3_)_3_•9H_2_O).

Conversion of PVC with bulk Ga LM beads yielded modest dechlorination (21%) and H_2_ yield (1.5%) (Figure 3a). In contrast, using Ga nitrate to provide exclusively Ga cations resulted in nearly complete dechlorination at 200 °C, although no H_2_ was generated (Figure 3a). Postreaction energy-dispersive X-ray spectroscopy (EDS) mapping showed that Ga remained well-dispersed after the reaction using Ga nitrate at 200 °C (Figure S6). The superior dechlorination attained using Ga nitrate was likely related to enhanced interaction with PVC. These findings suggested that while metallic Ga was indispensable for H_2_ production, cationic Ga more effectively promoted PVC dechlorination. Consequently, both Ga species can contribute to enhanced upcycling into dechlorinated carbon and H_2_.

Ab initio molecular dynamics (AIMD) supported the ability of metallic Ga to activate both C–Cl and C–H bonds in PVC (Figures S7 and S8). To investigate the interactions between PVC and liquid metallic Ga, two PVC chains, each consisting of six monomer units, were included in the simulations, which were performed at 900 K to enhance configurational sampling and accelerate polymer–metal interfacial dynamics. The simulations revealed substantial dechlorination as well as C–H activation of the PVC chains. Ten out of 12 chlorine atoms in the simulation cell detached from the polymer backbones in the simulation time frame (50 ps). During this time, we also observed three events of C–H bond breaking: two followed by H transfer to metallic Ga and one followed by H transfer to a neighboring C atom within the same PVC chain. Overall, these findings indicate that metallic Ga can facilitate both dechlorination and dehydrogenation, thus supporting our experimental observations.

After showing how Ga oxidation affects PVC dechlorination and H_2_ production, we prepared three batches of Ga LM flakes designed to have a higher proportion of cationic Ga (Figure S9). The three batches were characterized via X-ray photoelectron spectroscopy (XPS) and evaluated for catalytic performance (Figure 3b–d). Compared to bulk Ga LM beads, the Ga flakes offered a greater exposed surface area, leading to an increased ratio of oxidized Ga to metallic Ga upon exposure to ambient air. The three batches yielded a broad range of dechlorination (54–95%) and H_2_ production (1.6–7.2%) (Figure 3b). Surface analysis showed similar concentrations of fully oxidized Ga^3+^ and metallic Ga across all batches, with Ga^3+^ being predominant (Figures 3c, S10, and S11 and Table S1). However, the subsurface, revealed by Ar ion etching, displayed differing Ga species (Figure 3d). Notably, Batch III, which achieved the highest dechlorination (95%) and H_2_ yield (7.2%), did not present fully oxidized Ga^3+^ in the subsurface. Instead, the catalyst was dominated by partially oxidized Ga and metallic Ga. These results suggest that an optimal balance between Ga^0^ and oxidized Ga enhances both H_2_ yield and dechlorination efficiency. Although careful characterization of three selected batches of Ga flakes revealed insights into the importance of a controlled ratio of Ga^δ+^/Ga^0^ species in the catalyst, reproduction of Ga flake synthesis carries challenges due to the inhomogeneous size and shape of the flakes formed. Therefore, tailored and reproducible design of Ga^δ+^/Ga^0^ active sites in a core–shell architecture is presented next.

### Controlled Generation of Synergistic Ga^δ+^/Ga^0^ Active Sites

Thus, far, the synergistic role of Ga^δ+^/Ga^0^ species for PVC dechlorination and H_2_ production has been shown. Comparing the PVC upcycling performance achieved using bulk Ga LM beads and Ga nitrate showed that metallic Ga is indispensable for H_2_ production. Comparing the performance of Ga flakes showed that a balance of Ga sites with different oxidation states provides enhanced PVC dechlorination and H_2_ production. Now, the controlled synthesis of these dual sites is attained, and their tunability to boost dechlorination and H_2_ production rates is shown. Sonication of Ga LM in water^46–49^ was used to generate Ga-LMP, where a shell of GaOOH nanorods coated a Ga LM core, as characterized via electron microscopy and X-ray diffraction (XRD) (Figures 4 and S12). The core–shell structure of Ga-LMP, prepared by sonication of Ga LM beads during 1.5 and 3 h (samples labeled Ga-LMP-1.5 h and Ga-LMP-3 h, respectively), was characterized using plasma focused ion beam (PFIB) coupled with scanning electron microscopy (SEM) and energy-dispersive X-ray spectroscopy (EDS), as well as scanning transmission electron microscopy (STEM) coupled with EDS. PFIB was used to dissect Ga-LMP-1.5 h (Figure 4a) and Ga-LMP-3 h (Figure S13) samples after tilting the stage 52° for better visualization. The construction of a shell containing Ga and O and the existence of a Ga core was confirmed. STEM-EDS characterization of the Ga-LMP showed that sonication for 1.5 and 3 h created GaOOH shells of 136 ± 48 nm and 254 ± 73 nm thick, respectively (Figures 4b and Figure S14). Interestingly, Ga-LMP-1.5 h presented a thinner and more irregular shell, leading to sections where only a thin oxide layer covered the Ga LM core (see the red arrow in Figures 4b and S14a). Ga-LMP-3 h presented a thicker and more regular shell, and the core was covered on all particles characterized (Figures 4b and S14b).

**Figure 4:**
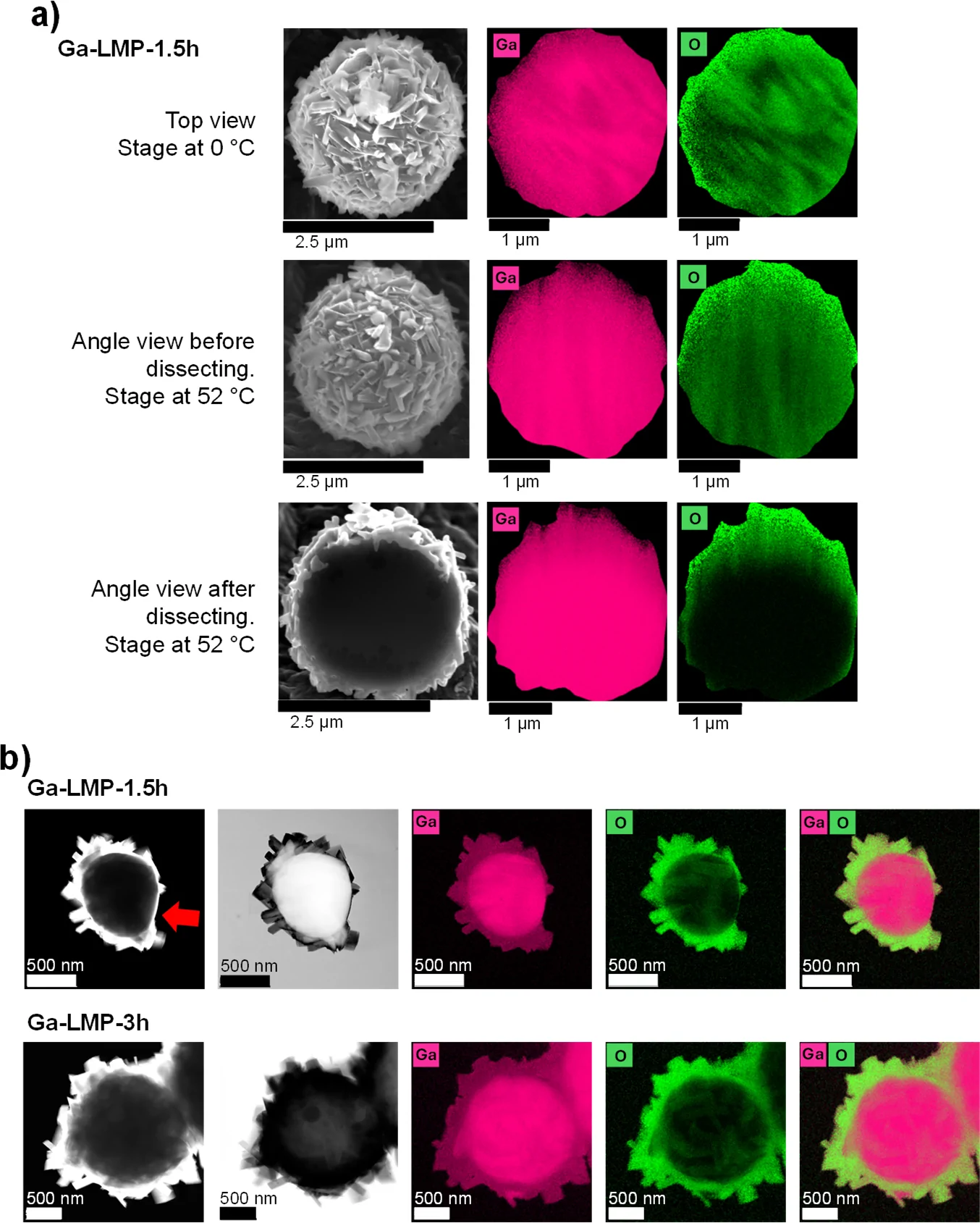
GaOOH-Ga core–shell structure was confirmed in Ga-LMP samples. (a) PFIB-SEM-EDS of Ga-LM-1.5 h. (b) STEM annular dark field, brightfield, and EDS mapping images of Ga-LMP-1.5 h and Ga-LMP-3 h. The red arrow shows an area where the Ga core was partially exposed.

The dechlorination and H_2_ yield attained by Ga-LMP-1.5h were superior to those attained by Ga-LMP-3 h at 150 and 160 °C (Figure 5a,b). Using Ga-LMP-1.5 h led to 62 and 98% dechlorination of PVC at 150 and 160 °C, respectively, whereas Ga-LMP-3 h did not attain dechlorination at 150 °C and attained only 24% dechlorination at 160 °C. The partial exposure of the Ga LM core through a GaOOH shell in Ga-LMP-1.5 h supports the synergistic interaction of dual Ga^δ+^/Ga^0^ sites to enhance PVC upcycling. H_2_ yield was negligible at 150 °C for both samples and reached similar values for both samples at 160 °C (Figure 5a,b).

**Figure 5:**
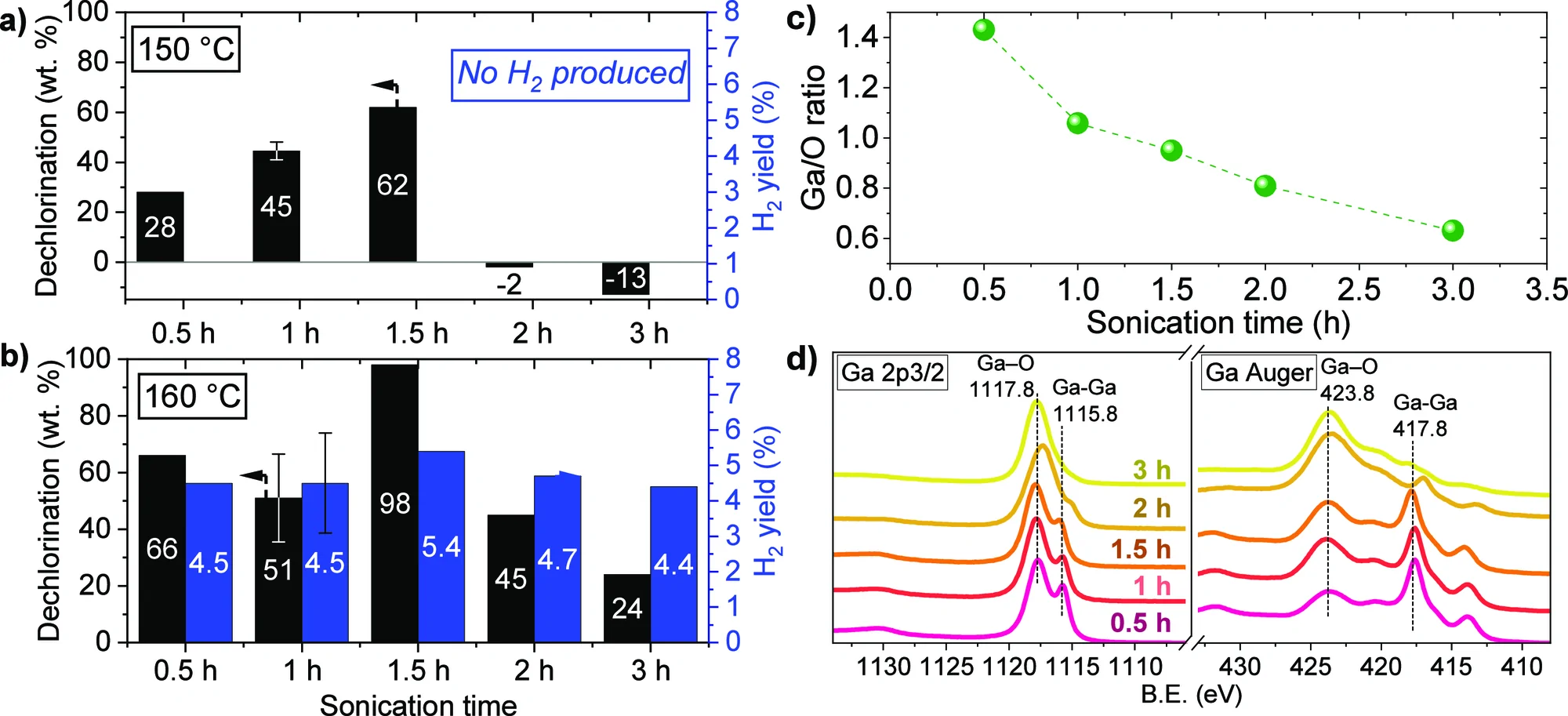
The longer the sonication time, the thicker the GaOOH shell in LMP. The thickness of the GaOOH layer impacts dechlorination and H_2_ yield. (a,b) PVC upcycling performance (at 150 and 160 °C) for Ga-LMP catalysts synthesized using different sonication times. Reaction conditions: 1 h, 0.2 g PVC, 0.049 g Ga-LMP. (c) Atomic ratio Ga/O according to XPS for Ga-LMP synthesized using different sonication times. (d) Ga 2p3/2 and Ga Auger XPS spectra of Ga-LMP synthesized using different sonication times.

Since the sonication time proved to greatly affect the catalytic performance of Ga-LMP, we studied the catalyst properties and catalytic performance of Ga-LMP catalysts synthesized using different sonication periods between 0.5 and 3 h. Tuning the sonication time allowed us to control the shell thickness of the particles, which is correlated to the atomic Ga/O ratio (Figure 5c) and greater presence of oxidized Ga at the surface (Figure 5d). Longer sonication times increased the thickness of the GaOOH shell, supporting findings via STEM-EDS (Figures 4b and Figure S14). Sonication times between 1 and 1.5 h provided the greatest dechlorination at 150 °C (Figure 5a). A sonication time of 1.5 h provided the highest dechlorination and H_2_ yield at 160 °C (Figure 5b).

During the reaction, the thin GaOOH shell of Ga-LMP-0.5 h and Ga-LMP-1 h catalysts was removed through interactions with HCl, which facilitated the recovery of the Ga LM core, given that it naturally separates from the dechlorinated solid product. The easy recovery of the Ga core enabled catalyst reusability. Given its catalytic performance and ease of recovery, Ga-LMP-1 h was selected for further studies.

To evaluate the reusability of the Ga-LMP-1 h catalyst, the catalyst was recycled multiple times and used to catalyze the conversion of fresh PVC at 160 °C (Figure S15a). For the initial run, 0.2 g of Ga-LMP-1 h in the powder form was used. At the end of the run, the GaOOH shell in Ga-LMP-1 h was removed, leaving a Ga LM bead behind (Figure S15b). For the reusability tests, the Ga LM bead was recovered, placed in an empty reactor, and resonicated for 1 h to reconstruct the GaOOH shell, followed by drying. Resonication inside the reactor generated Ga-LM-1 h powder, but a smaller Ga LM bead remained. This mixture of Ga-LM-1 h and a smaller Ga LM bead was labeled Ga-LMP-1 h*. After the initial run with Ga-LMP-1h, Ga-LM-1 h* presented reduced dechlorination and H_2_ yield. The theoretical maximum dechlorination through stoichiometric GaCl_3_ trapping was calculated for each cycle. Figure S15 shows that the dechlorination attained by Ga-LM-1 h* after four cycles surpassed the maximum conversion attained if the conversion was stoichiometric, confirming the catalytic nature of the process. The amount of Ga-LM-1 h* loaded in the reactor was lower after each cycle (initial amount of Ga-LM-1 h = 0.2 g, amount of Ga-LM-1 h* loaded in each cycle = 0.078, 0.056, 0.035, 0.029 g; corresponding to a Ga-LM1-h* loss in each cycle of = 61, 28, 38, 17%). Since GaCl_3_ can coexist in HCl/water mixtures,^50^ we evaluated whether Ga was leaching into the water washes. ICP characterization of the first wash after conducting PVC upcycling for 1 h at 160 °C using 0.049 g of Ga-LMP-1 h revealed that the amount of Ga was no greater than 3 mg in the 30 mL water wash. This corresponds to a 6% catalyst lost. Thus, it was concluded that the loss of the catalyst during the reusability experiments was mainly due to limitations in the manual recovery of Ga. Optimizing mechanical recovery of the catalyst and sonication conditions for different amounts of Ga lies beyond the scope of this work.

#### Reaction Mechanism

The reaction mechanism for PVC upcycling over Ga-LMP-1 h was investigated by conducting the reaction at different temperatures for 1 h. Afterward, the gas overhead, solid product, and liquid product (acetone solutions) were collected for analysis. The dechlorination results indicated that heating to at least 130 °C was necessary to achieve any dechlorination of PVC under the reaction conditions studied. As the temperature increased, so did the extent of dechlorination. The process reached 87% dechlorination at 170 °C and 99% at 200 °C. H_2_ production was observed only at temperatures of 160 °C or higher (see Figure 6a). NMR analysis of the acetone wash confirmed production of mesityl oxide and diacetone alcohol in the temperature range 160–200 °C, which was generated by the HCl-catalyzed conversion of acetone (Figure S16).

**Figure 6:**
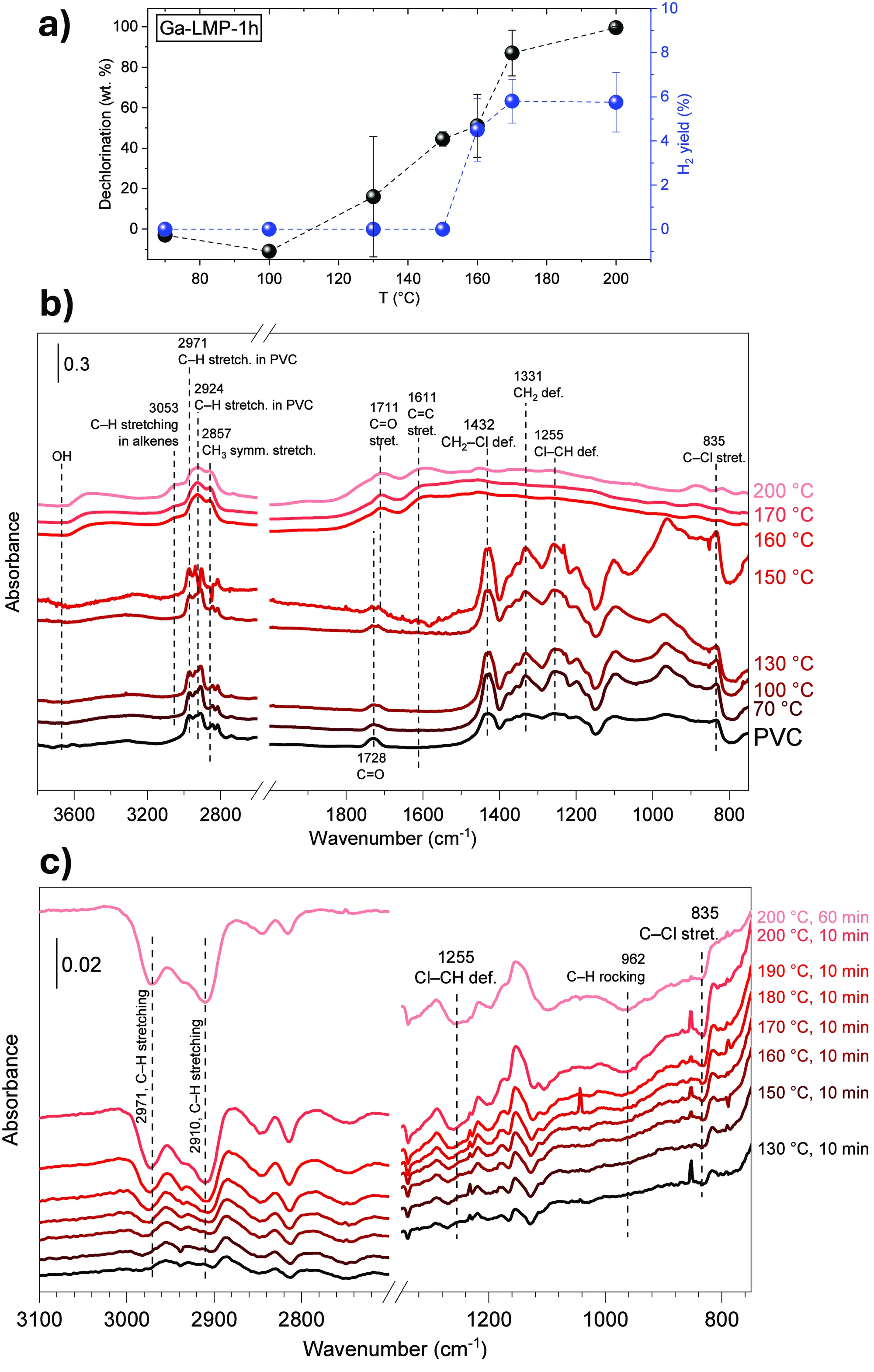
PVC dechlorination has a lower onset temperature than H_2_ production. Chain scission, H_2_ production, and the formation of double bonds start at 160 °C. (a) PVC dechlorination and H_2_ yield as a function of temperature using Ga-LMP-1 h. Reaction conditions: 1 h, 0.2 g PVC, 0.049 g Ga-LMP-1 h. (b) Ex situ DRIFTS of solid products. Reaction conditions: 1 h, 0.2 g PVC, 0.049 g Ga-LMP-1 h. Background: 25 °C, Ar atmosphere, KBr. (c) In situ DRIFTS using a 1:1 (wt/wt) mixture of PVC:Ga-LMP-1 h. Temperature was increased stepwise and held for 10 min at 130, 150, 160, 170, and 180 °C and at 200 °C for 1 h. Background: 25 °C, Ar atmosphere, mixture 1:1 (wt) of PVC and Ga-LMP-1 h.

Ex situ DRIFTS spectra of the solids produced at temperatures between 70 and 130 °C closely resembled that of pure PVC (Figure 6b). However, at 150 °C, a noticeable transition occurred in the spectrum. At temperatures of 160 °C and above, the IR spectra deviated substantially from that of the original PVC. Vibrational modes related to chlorinated carbon became indistinguishable (1432, 1255, 835 cm^–1^).^51–56^ However, at 160 °C, the threshold temperature for H_2_ production, the IR spectrum of the solid product exhibited three distinct changes: (1) a new vibrational mode at 2857 cm^–1^ appeared, corresponding to the symmetric stretching of CH_3_;^15^ (2) a band at 1611 cm^–1^ emerged, indicating the formation of unsaturated C=C bonds;^16,53^ (3) a shoulder at 3053 cm^–1^ emerged corresponding to C–H stretching in alkenes;^15^ and (4) the carbonyl vibration (present likely due to photodegradation during storage),^57^ originally observed at 1728 cm^–1^ in fresh PVC,^52,55,56^ red-shifted to 1711 cm^–1^, supporting the presence of an adjacent C=C bond.^58^ These observations implied that chain scission, H_2_ production, and the formation of double bonds occurred concurrently at higher temperatures (≥160 °C). This mechanism is consistent with the observations from Ga nitrate-catalyzed PVC conversion: despite the high dechlorination of PVC, no H_2_ was produced and features of unsaturated bonds were not present in the IR spectrum of the solid product (Figure S17).

To complement ex situ DRIFTS analysis, in situ DRIFTS was also performed. A 1:1 (wt/wt) mixture of PVC:Ga-LMP-1h was loaded in the IR cell. The sample was heated up to 200 °C under Ar, and spectra were recorded (Figure 6c). Although this in situ setup lacks stirring of the mixture, observations supported conclusions drawn from ex situ DRIFTS experimentation. At temperatures as low as 150–160 °C, features of dechlorination start to appear (C–Cl stretching at 835 cm^–1^). The decrease of C–H stretching features in PVC (2971 and 2910 cm^–1^) becomes more significant as the temperature increases, suggesting that the backbone of the PVC chain changed significantly during upcycling. Features regarding unsaturation of the solid product were not evident (Figure S18), and H_2_ production was not detected via online MS monitoring. These results suggest that the in situ setup did not effectively promote exposure of the metallic Ga core, which facilitates H_2_ production via C–C bond unsaturation, likely due to limited mass transfer.

To gain further insight into the chemical structure of the solid product, ^13^C magic-angle spinning (MAS) NMR spectroscopy characterization of the solid product was performed. Characterization of the solid product obtained when the reaction was conducted at 130 and 150 °C revealed two distinct peaks corresponding to −CHCl– (55 ppm) and linear −CH_2_– (45 ppm), which correspond to carbon atoms present in fresh PVC.^59,60^ Starting at 160 °C, features corresponding to aromatic (∼130 ppm) and cyclic −CH_2_– (∼30 ppm) groups appeared in the solid products (Figure 7a,b).^61^ Interestingly, linear −CH_2_– (45 ppm) functionalities gradually disappeared from the solid product as the reaction temperature was increased, whereas cyclic −CH_2_– functionalities remained relatively stable between 160 and 200 °C, and aromatic features increased gradually with temperature. This can be rationalized through the dechlorination of PVC, followed by chain cross-linking to generate aliphatic cyclic structures, which later dehydrogenated to produce H_2_ gas and a polyaromatic structure. The relatively constant concentration of cyclic −CH_2_– between 160 and 200 °C supported an intermediate cyclic aliphatic structure between the linear −CH_2_– in PVC and aromatic functionalities. To further characterize the molecular motions and functionalities of the solid product obtained after conducting the reaction at 200 °C, inelastic neutron scattering (INS) experimentation was performed. Figure 7c shows that the solid product had suppressed molecular motions (∼400–1000 cm^–1^) compared to the PVC molecule, supporting the formation of a more rigid cyclic structure. Further, suppression of vibrational modes corresponding to the −CH_2_– angle change and stretching, as well as hydrogen vibrations in −CHCl–, supports the low chlorine content and more rigid nature of the solid product.

**Figure 7:**
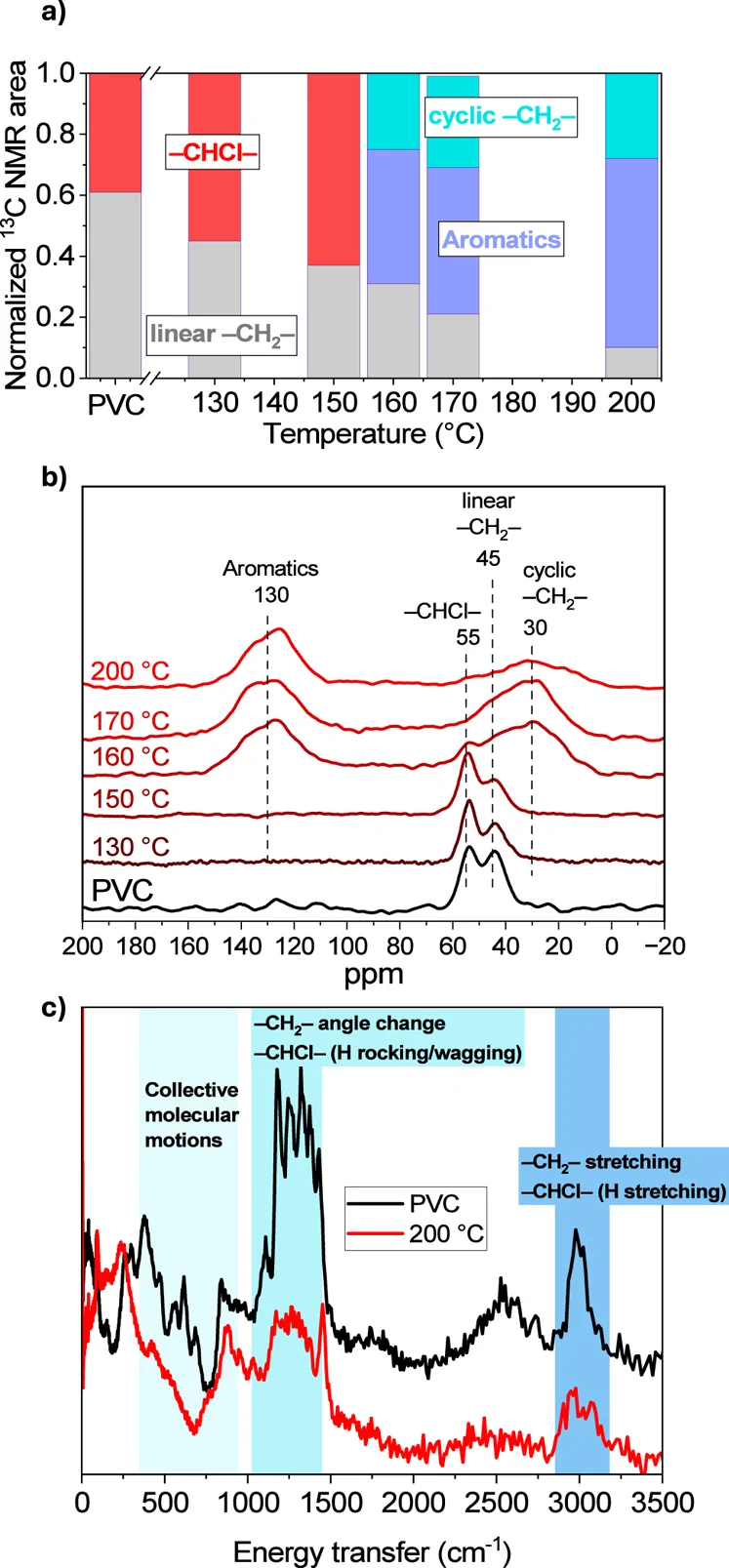
Cyclic compounds are formed starting at 160 °C. Greater temperatures increase the aromaticity of the solid. (a) Relative ratios of functional groups and (b) ^13^C MAS NMR spectra of PVC and solid products. (c) INS spectra of fresh PVC and the carbon product obtained at 200 °C. Reaction conditions: 1 h, 0.2 g PVC, 0.049 g Ga-LMP-1 h

Collectively, characterization of the Ga-LMP catalyst and the gas, solid, and liquid products allowed us to portray a reaction mechanism as presented in Figure 8. First, the PVC chain interacted with the GaOOH shell of the Ga-LMP catalyst, where cationic Ga initiated dechlorination of the PVC chain to produce H^+^ and Cl^–^ ions adsorbed on the GaOOH surface. Next, the dechlorinated PVC chains cross-linked to generate a polycycloalkane product, while concurrently H^+^ ions reduced the GaOOH shell to expose the Ga^0^ core. The Ga^0^ core promoted dehydrogenation of the solid product, leading to partial unsaturation of the structure and generating hydrogen gas. After the reaction, the Ga^0^ core is left mostly exposed. The GaOOH shell can be reformed by regenerating the catalyst via sonication. It is worth noting that the steps in the reaction mechanism shown in Figure 8 may occur simultaneously. At present, the rate-limiting step is unclear—it could involve dehydrogenation of cyclized intermediates, exposure of the Ga core, or the mass transfer/contact between the evolving polymer structure (transitioning from linear to cyclic to aromatic) and the Ga active sites. In fact, evaluating dechlorination and H_2_ production as a function of reaction time suggests that multiple steps of the mechanism occur concurrently (Figure S19).

**Figure 8:**
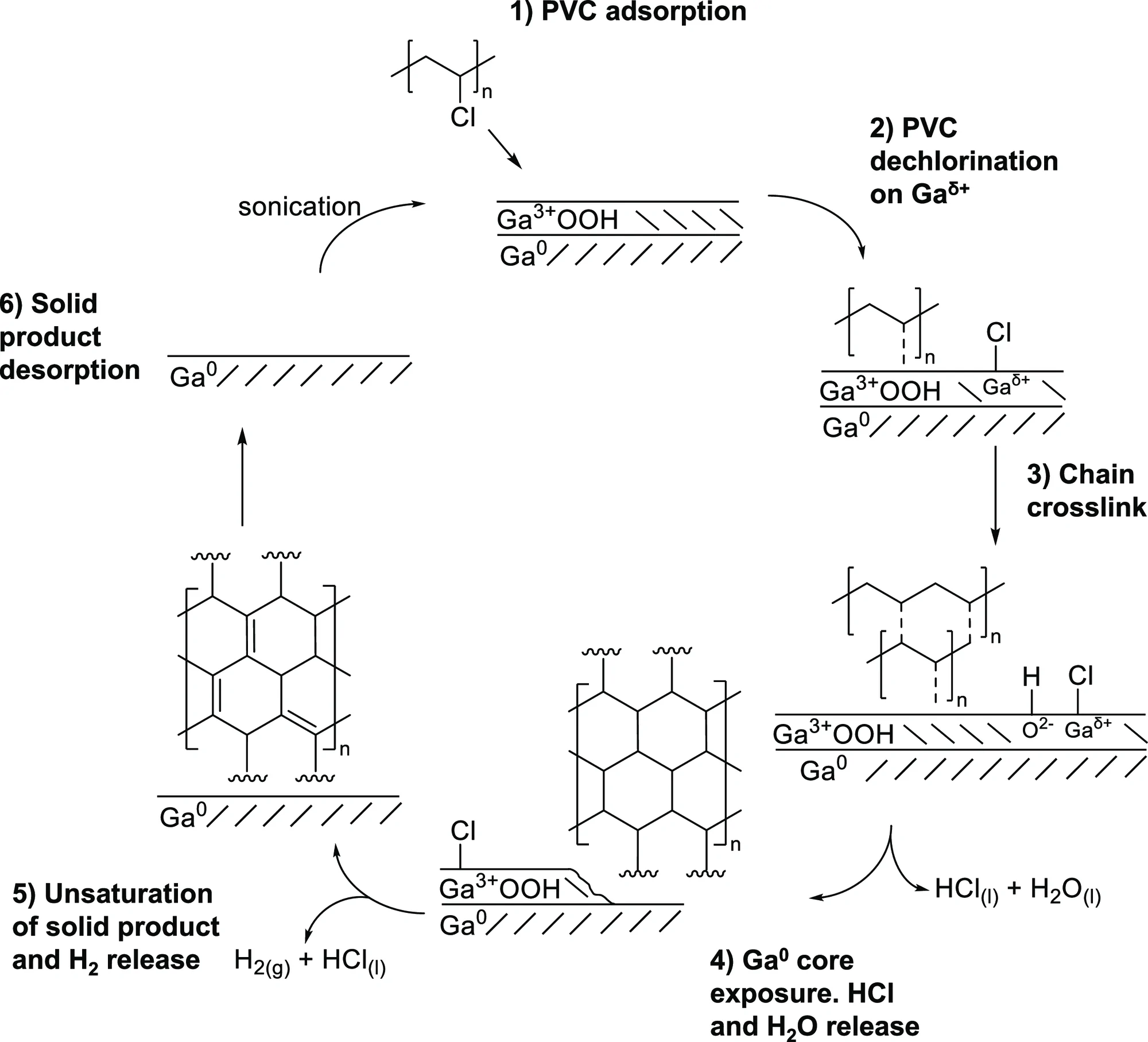
Proposed reaction mechanism for PVC upcycling over Ga-LMP-1 h. Dechlorination occurs first on cationic Ga. Next, H_2_ is produced through unsaturation of the cyclic product over Ga^0^.

## Conclusion

This work demonstrated a catalytic strategy for the low-temperature upcycling of polyvinyl chloride (PVC) using gallium liquid metal particles (Ga-LMP) with a dynamic Ga-GaOOH core–shell architecture. At 160 °C, this approach overcomes the common challenges associated with PVC recycling, namely, its thermal instability and nonmelt behavior, to deliver a highly dechlorinated carbonaceous solid, aqueous HCl, and gas-phase H_2_. Mechanistic investigations reveal that the success of this approach lay in the unique dynamic cooperation of Ga^δ+^/Ga^0^ active sites in the Ga-GaOOH core–shell configuration of the particles. Cationic Ga sites initiate C–Cl bond activation, while progressive reduction of the shell exposes metallic Ga sites that promote C–H activation to produce H_2_. Tuning the shell thickness of the catalyst led to a core–shell structure where the core was easily exposed during the reaction, which later facilitated catalyst separation from the dechlorinated solid product. The GaOOH shell of the original Ga-LMP catalyst can be then regenerated via resonication of the exposed Ga core. This dynamic dual-site synergism provides fundamental principles for novel catalyst design to drive complex chemical transformations involving activation of multiple bond types for efficient upcycling of recalcitrant plastics.

## Materials and Methods

### Materials

PVC (average molecular weight (*M*_w_) 233,000, average number-average molecular weight (*M*_n_) 99,000), gallium nitrate hydrate (99.9), acetone (≥99.5%), and CDCl_3_ (1% (v/v) TMS) were sourced from Sigma-Aldrich. Gallium (99.9%) was sourced from Thermoscientific. Phenolphthalein solution was purchased from Sigma-Aldrich (0.5 wt % in ethanol/water (1:1)).

### Catalyst Synthesis

#### Ga Flake Synthesis

Beads of Ga (<0.5 g) were placed in a closed 20 mL quartz vial, and the vial was heated to 40–60 °C in warm water to liquefy Ga. Next, the vial was vigorously shaken in all directions. After shaking, the vial was placed in a refrigerator at 6 °C for approximately 10 min. After that, the vial was removed from the refrigerator, and flakes were peeled off the interior of the vial (mostly from the lid). Flakes were placed into a new vial and stored in the refrigerator.

#### Ga-LMP Synthesis

A Fisher FS60H sonicator (42 kHz ±6%) was preheated to 58–64 °C with an adequate water level. Ga beads and deionized (DI) H_2_O in a ratio of 0.15 g Ga: 1 mL DI H_2_O were placed in a 20 mL quartz vial. The glass vial was then placed in the middle of the sonicator pool. The vial was halfway submerged, and the lid was slightly ajar. The mixture was sonicated for different periods of time between 0.5 and 3 h before the vial was removed from the sonicator pool. The content of the vial was then swirled to mix particles inside the vial. After swirling, the vial was placed in a vacuum oven at 95 °C for 1.5 h or until dry. After drying, the Ga-LMP was stored in a cool dry place. For sonication times of 1.5 h or less, a bead of LM and the Ga-LMP were present in the vial. The bead was discarded, and the Ga-LMP was used for all tests, except for the catalyst reusability test, as explained in the next section.

### Catalytic Conversion and Gas Overhead Characterization

A pressure-rated glass tube reactor of approximately 38 mL volume and 20 cm length alongside a 12.7 × 5 mm inert glass-coated stir bar was used for the catalytic conversion. The required amounts of Ga (beads, flakes or LMP) and PVC (presieved to 177–250) μm were added to the reactor.

When Ga nitrate (Ga­(NO_3_)_3_•9H_2_O) was used, the desired amount of Ga­(NO_3_)_3_•9H_2_O was dissolved in 1–2 mL of DI H_2_O in the glass reactor. Then, the desired amount of PVC was added. The mixture was swirled and then placed in an oven at atmospheric pressure. The mixture was dried for at least 3 h at 95 °C.

After loading the reactor with Ga (beads, flakes, LMP, or Ga nitrate) and PVC, the reactor was flushed with 50 mL/min of Ar to create an inert gas atmosphere, prior to conducting the batch catalytic conversion. Next, the reactor was placed in a preheated oil bath, and the reaction was allowed to proceed for the desired time under 1200 rpm of stirring. After the reaction finished, the reactor was cooled by submerging it in water at room temperature. The reactor gas overhead was sampled by withdrawing gas with a needle using a septum placed in the overhead space. The gas was analyzed by using a ThermoStar Pfeiffer mass spectrometer (MS). The MS signal was calibrated with known concentrations of the gas mixtures. Product selectivity in the gas phase was estimated by comparing the calibration of MS signals and the pressure of the gas overhead, which was monitored with a pressure gauge.

The solid reacted material was removed from the reactor, and any visible spent Ga was separated. The solid reacted material was washed with 30 mL of water (or acetone) and centrifuged at 10,000 rpm for 5 min; the wash–centrifuge cycles were repeated six times. The solid was dried overnight at 95 °C.

#### Catalyst Reusability Tests

Catalyst reusability tests were performed on Ga-LMP-1 h. For the initial run, 0.2 g of Ga-LMP-1 h in the powder form was used. For the reuse tests, the Ga LM bead recovered was placed in the 38 mL reactor and sonicated for 1 h at 58–64 °C in DI water (0.15 g Ga/ml DI water). The reactor was submerged 5 cm into the sonication bath. Next, the reactor was placed in a vacuum oven at 95 °C for 1.5 h or until dry. All contents inside the reactor were used for the next catalytic run; therefore, the catalyst used in the reuse runs consists of a Ga LM bead and Ga-LMP powder, and this catalyst is labeled as Ga-LMP-1 h*. An amount of 0.2 g of fresh PVC was used for each reuse run.

### Catalyst Characterization

#### X-ray Photoelectron Spectroscopy (XPS)

XPS was performed using a Thermo Scientific (Waltham, MA, USA) Model Nexsa G2 XPS instrument. The instrument utilizes monochromatic, microfocused, Al K_α_ X-rays (1486.6 eV) with a variable spot size (i.e., 30–400 μm). Typically, a 400 μm X-ray spot size is used for the maximum signal and to characterize the largest possible area. The instrument has a hemispherical electron energy analyzer equipped with a 128-channel detector system. Vacuum in the analysis chamber is at least 2 × 10^–9^ mbar. Data were collected and processed using the Thermo Scientific Avantage XPS software package (v.6.8.1). Three batches of Ga flakes were analyzed. Both sides of a representative flake from each batch were characterized. After examining the as-received surfaces, a 30 s Ar-ion etch was done to characterize the subsurface. According to calibration of the ion gun parameters at a sputter rate of 15 nm/min on standard SiO_2_ films, assuming the Ga flake surface sputtered at the same rate, 30 s of etching removed ∼7–8 nm of the outer surface. After 30 s of etching, spectra were acquired. In addition, five samples of Ga-LMP were analyzed. These particles were prepared for analysis by dispersing onto double-sided tape.

#### X-ray Diffraction (XRD)

XRD of Ga-LMP was measured using a PANalytical X′Pert Pro MPD equipped with an X′Celerator solid-state detector (Cu Kα source, λ = 0.1542 nm, 45 kV, 40 mA). The 2θ scan ranged from 5° to 90°.

#### Microscopy

Scanning transmission electron microscopy (STEM) combined with energy-dispersive X-ray spectroscopy (EDS) elemental mapping was conducted using an FEI Talos F200X STEM operated at 200 kV and equipped with four silicon drift X-ray detectors. Spectrum images were acquired at a resolution of 1024 × 1024 pixels with a dwell time of 50 μs per pixel. Quantitative EDS analysis was performed using TFS Velox software. For STEM imaging, the collection angle was 0–9 mrad for bright-field (BF) imaging and 61–200 mrad for annular dark-field (ADF) imaging. Plasma focused ion beam (PFIB) milling operations, scanning electron microscopy (SEM) imaging, and EDS elemental mapping were performed using a Thermo Scientific Helios Hydra PFIB-SEM. Secondary electron imaging and EDS mapping were performed at 10 kV to minimize sample charging. Elemental maps were collected using an Oxford UltimMax 170 detector with a dwell time of 15 μs per pixel and 50 frames per map. Quantitative EDS analysis was performed using AZtecLive software. Sample sectioning was carried out using a xenon beam at 30 kV with a beam current of 0.1 nA.

### Solid Product Characterization

#### Thermogravimetric Analysis-MS (TGA-MS) of the Carbonaceous Material

TGA-MS was performed in a TA TGA Q5000 system, and the combustion/decomposition products were monitored using a ThermoStar Pfeiffer MS. Approximately 5 mg of the reacted, washed, and dried carbon product was added to a tared TGA pan and heat-treated under 100 mL/min of synthetic air. The heat-treatment consisted of an initial isothermal treatment at 45 °C for 30 min, followed by a ramp to 750 °C at 10 °C/min. The extent of dechlorination was calculated by comparing HCl released from the solid product sample with HCl released from fresh PVC. All dechlorination values reported are in a per-weight basis. A negative dechlorination means that atoms other than Cl were removed from the solid product, making its Cl content greater than that of fresh PVC.

$$dechlorination=100\times \frac{\left[Cl\;content\;in\;fresh\;PVC\right]-\left[Cl\;content\;in\;the\;solid\;product\right]}{\left[Cl\;content\;in\;fresh\;PVC\right]}$$

#### Diffuse Reflectance Infrared Fourier Transform Spectroscopy (DRIFTS)

Ex situ analyses of the samples were performed using a Nicolet Nexus 670 FTIR spectrometer. A background spectrum with KBr under Ar at room temperature was used. Each product sample was mixed with KBr (10 g KBr: 1 g sample) and loaded into a crucible, which was placed in a DRIFTS cell (Pike Technologies). First, a KBr–PVC mixture was pretreated at 95 °C for 1 h under Ar and then cooled down to 25 °C. A reference spectrum was collected. Next, KBr–product mixtures were loaded in the DRIFTS cell, pretreated at 95 °C for 1 h under Ar, and then cooled down to 25 °C. IR spectra were collected. For in situ analysis, a background at room temperature under Ar for a mixture 1:1 (wt) of PVC and Ga-LMP-1 h was collected. Next, temperature was ramped (1 °C/min) stepwise and held for 10 min at 130, 150, 160, 170, and 180 °C and at 200 °C for 1 h.

#### Solid-State Magic-Angle Spinning (MAS) Nuclear Magnetic Resonance (NMR)

MAS NMR characterization was conducted on a 700 MHz Tecmag Redstone NMR spectrometer. The spectrometer features a triple-resonance 3.2 mm probe. A Hahn echo refocusing over a single rotor period of spinning at 10 kHz was used for direct detection experiments.

#### Inelastic Neutron Scattering (INS)

INS data were collected at the VISION beamline of the pulsed Spallation Neutron Source (SNS) of Oak Ridge National Laboratory. VISION is a backscattering spectrometer. Samples were exposed to a white neutron beam, and the final energy of the scattered neutrons was fixed by Bragg reflection on pyrolytic graphite analyzers. An amount of 0.4 g of each sample was loaded in a V can (6 mm), and the measurement was performed at approximately 5 K. Data were reduced using the software Mantid.^62^

### Liquid Product Characterization

#### Titration Using Phenolphthalein

After washing and centrifuging the carbon-based product, the resulting water wash was combined with a phenolphthalein indicator. A buret containing 0.1 M aqueous NaOH was then used to titrate the mixture, with the base added slowly while stirring at 700 rpm. The titration was considered complete when the solution turned pink, indicating that all of the chlorine had been titrated.

#### Inductively Coupled Plasma–Optical Emission Spectroscopy (ICP-OES)

Gallium content of aqueous solutions was determined using ICP-OES in the radial mode using an iCAP 7000 spectrometer (Thermofisher Scientific) equipped with a prepFast M5X intelligent dilution automatic sample loader (Elemental Scientific, Omaha, Nebraska). Experimental solutions were filtered through syringe filters with a 0.45 μm pore size.

#### Ab Initio Molecular Dynamics (AIMD) Simulations

Ab initio molecular dynamics (AIMD) simulations were performed using the Vienna Ab initio Simulation Package (VASP) within the framework of density functional theory (DFT).^63,64^ The projector augmented-wave (PAW) method was used to describe the ion–electron interactions, and the exchange–correlation energy was treated using the Perdew–Burke–Ernzerhof (PBE) generalized gradient approximation (GGA).^63,65,66^ A plane-wave kinetic energy cutoff of 500 eV was employed. Dispersion interactions were included via the DFT-D3 method.^67^ Brillouin-zone integrations were carried out at the Γ-point only. AIMD simulations were performed in the canonical (*NVT*) ensemble using a Nosé–Hoover thermostat.^68,69^ The equations of motion were integrated with a time step of 1 fs. Periodic boundary conditions were applied in all directions. The molten structure of liquid Ga was generated using AIMD simulations at 480 K. To investigate the interactions between PVC and liquid Ga, two PVC chains, each consisting of six monomer units, were included for the geometry optimization. Then, *NVT* simulations were performed at 900 K to enhance configurational sampling and accelerate polymer–metal interfacial dynamics. The simulations were run for 50 ps.

## Supplementary Material



## Data Availability

This manuscript has been authored by UT-Battelle, LLC under Contract No. DE-AC05–00OR22725 with the U.S. Department of Energy. The United States Government retains and the publisher, by accepting the article for publication, acknowledges that the United States Government retains a nonexclusive, paid-up, irrevocable, worldwide license to publish or reproduce the published form of this manuscript, or allow others to do so, for United States Government purposes. The Department of Energy will provide public access to these results of federally sponsored research in accordance with the DOE Public Access Plan (http://energy.gov/downloads/doe-public-access-plan).

## Notes

Gas overhead compositions, ^1^H and ^13^C NMR spectra, photographs of silver nitrate test, dechlorination of the carbonaceous product, STEM and EDS images, AIMD models and description of bond breaking events, photographs of fresh and spent catalysts, XPS spectra and fitting, XRD spectra, PFIB-SEM-EDS images, and IR spectra (PDF)
