# Supplementary material for: Enhanced Catalytic Dechlorination of Polyvinyl Chloride (PVC) and H2 Production Enabled by Synergistic Gaδ+/Ga0 Active Sites in Liquid Metal Particles
Source: J Am Chem Soc. 2026 Sep 2;148(36):38797–809. doi: 10.1021/jacs.6c09418 (PMC13587679; doi:10.1021/jacs.6c09418)
Supplement: Supplementary file 1 [file ja6c09418_si_001.pdf]

Enhanced Catalytic Dechlorination of Polyvinyl Chloride (PVC) and H_2 Production Enabled by Synergistic $Ga^{\delta+}/Ga^0$ Active Sites in Liquid Metal Particles

Benjamin H. Crockett Zingg[1][2], Nabihan B. Abdul Rahman[2], Nishu Devi[2], Anya Zornes[2], Joshua T. Damron[2], Ernesto C. Zuleta[2], Yan-Ru Lin,[3] José David Arregui-Mena,[3] Chang Liu[2][4], Murillo Longo Martins[4], Shukai Yao[5], Mo Li[5], De-en Jiang[5], Harry Meyer III[2], Zili Wu[2], Felipe Polo-Garzon[2]*

[1] Department of Chemistry, Kentucky State University. Frankfort, KY 40601 (USA)
[2] Chemical Sciences Division, Oak Ridge National Laboratory. Oak Ridge, TN 37831 (USA)
[3] Materials Science and Technology Division, Oak Ridge National Laboratory, Oak Ridge, TN 37831 (USA)
[4] Neutron Scattering Division, Oak Ridge National Laboratory, Oak Ridge, TN 37831 (USA)
[5] Chemical and Biomolecular Engineering Department, Vanderbilt University, Nashville, TN 37235 (USA)

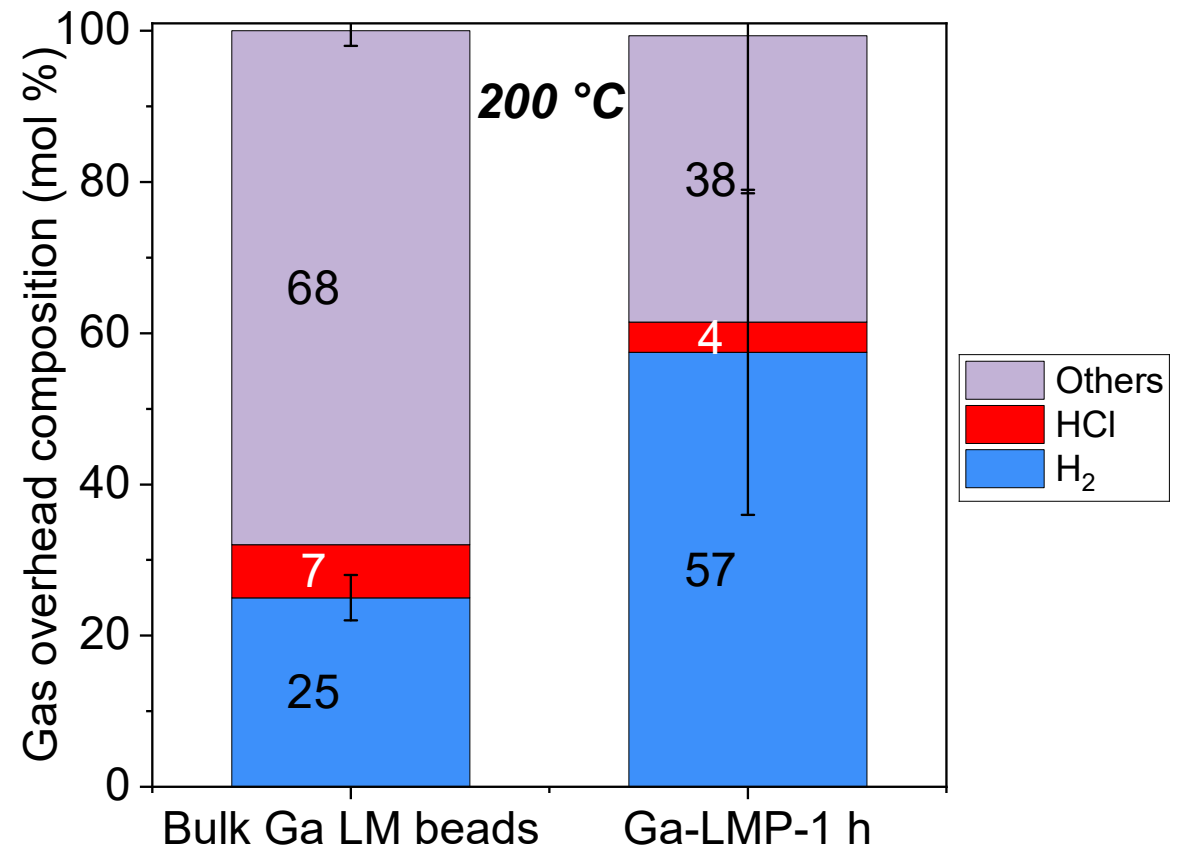

Figure S1. Composition of the gas overhead after 1 h reaction, using PVC (Sigma Aldrich) and either bulk Ga LM beads or Ga-LMP-1h as catalysts. *Reaction conditions: 1 h, 200 °C, 0.2 g PVC, 0.049 g of catalyst.*

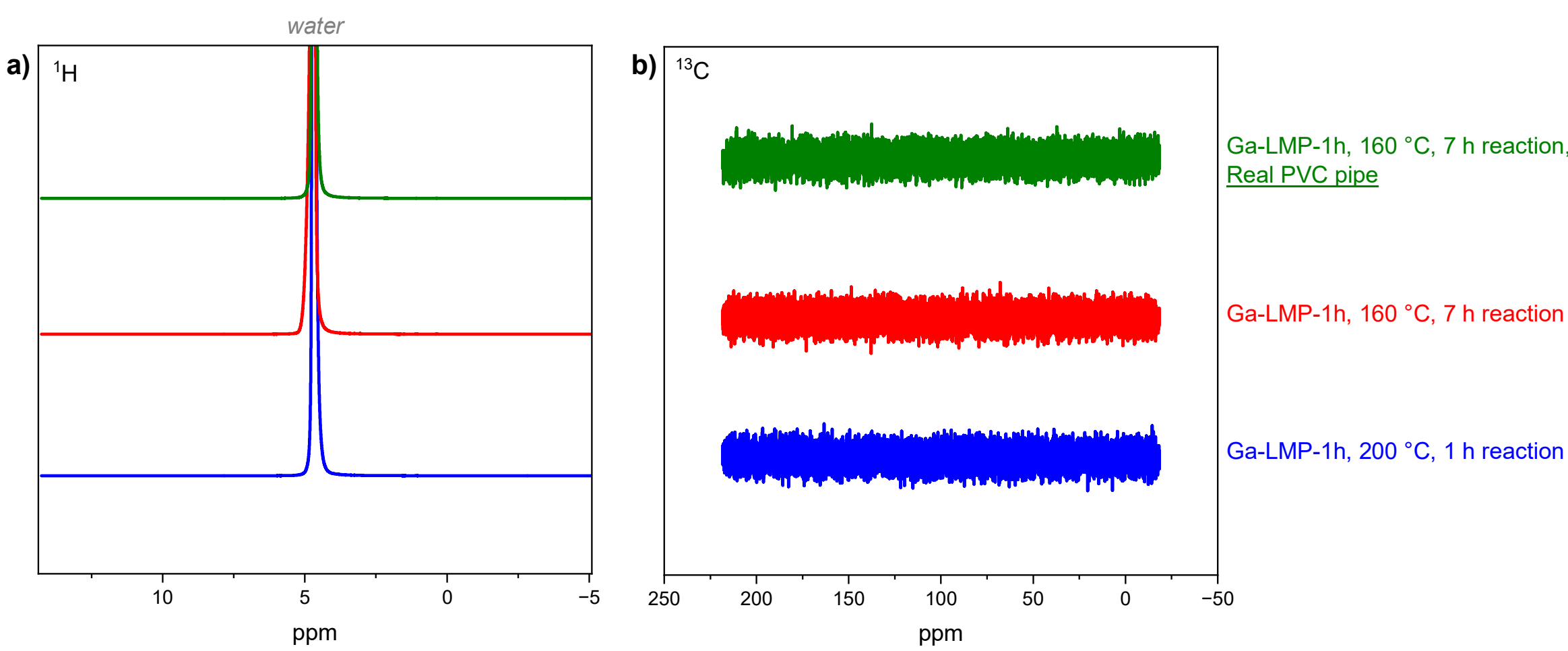

Figure S2. Liquid ^{1}H (**a**) and ^{13}C NMR (**b**) of water washes collected after PVC upcycling using Ga-LMP-1h catalyst at various conditions. An amount of 0.2 g PVC and 0.049 g catalyst were used for all runs. All solutions analyzed were mixed with equal volume of D_2O before NMR characterization.

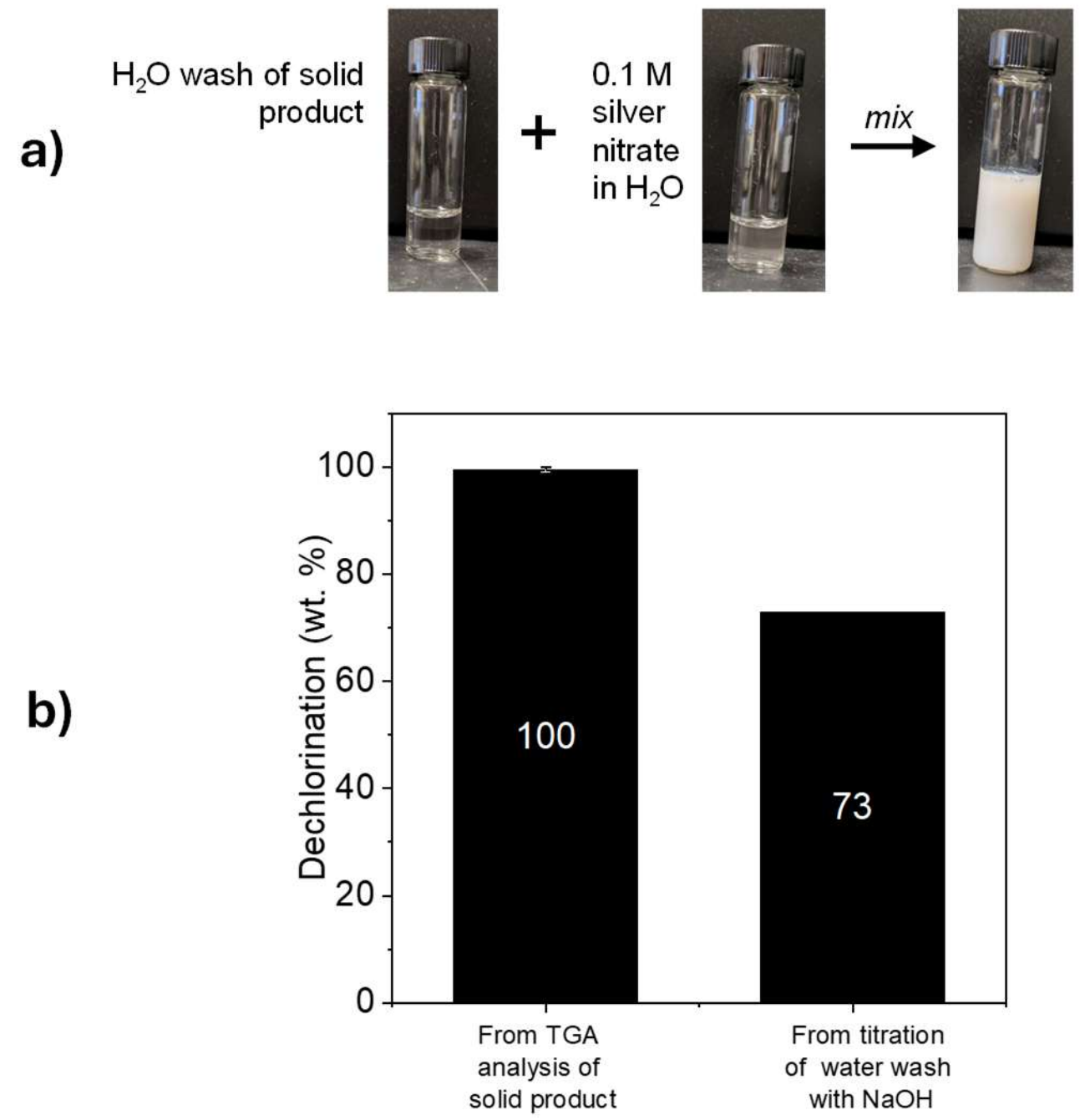

Figure S3. a) Representative silver nitrate test to identify Cl^- in 1st H_2O wash of dechlorinated solid product after upcycling PVC using Ga LM-based catalyst. Formation of white precipitate (AgCl) confirms the presence of Cl^- in the wash solution. **b)** Comparison of dechlorination estimated from Cl content in the solid carbon product (via TGA-MS) and dechlorination estimated from titration of the water wash using NaOH and phenolphthalein as indicator (*Reaction conditions: 1 h, 0.2 g PVC, 0.049 g Ga-LMP-1h*).

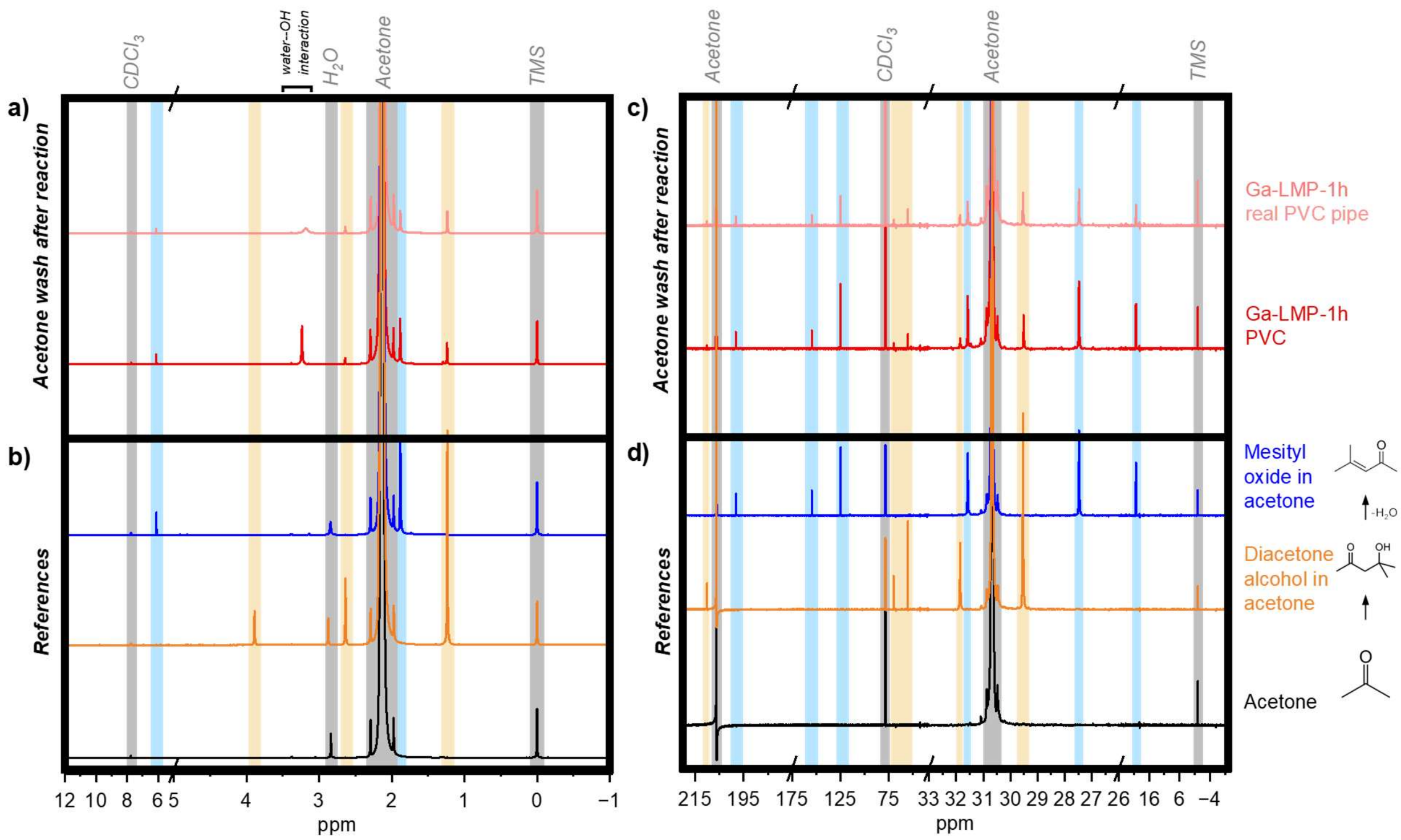

Figure S4. Liquid ^{1}H (**a,b**) and ^{13}C NMR (**c,d**) of: **a,c)** Acetone washes collected after reaction at different temperatures. *Reaction conditions: 6-7 h, 0.2 g PVC, 0.049 g Ga-LMP-1h*. **b,d)** Mesityl oxide, diacetone alcohol and acetone references. All solutions analyzed were mixed with equal volume of $CDCl_3$ before NMR characterization.

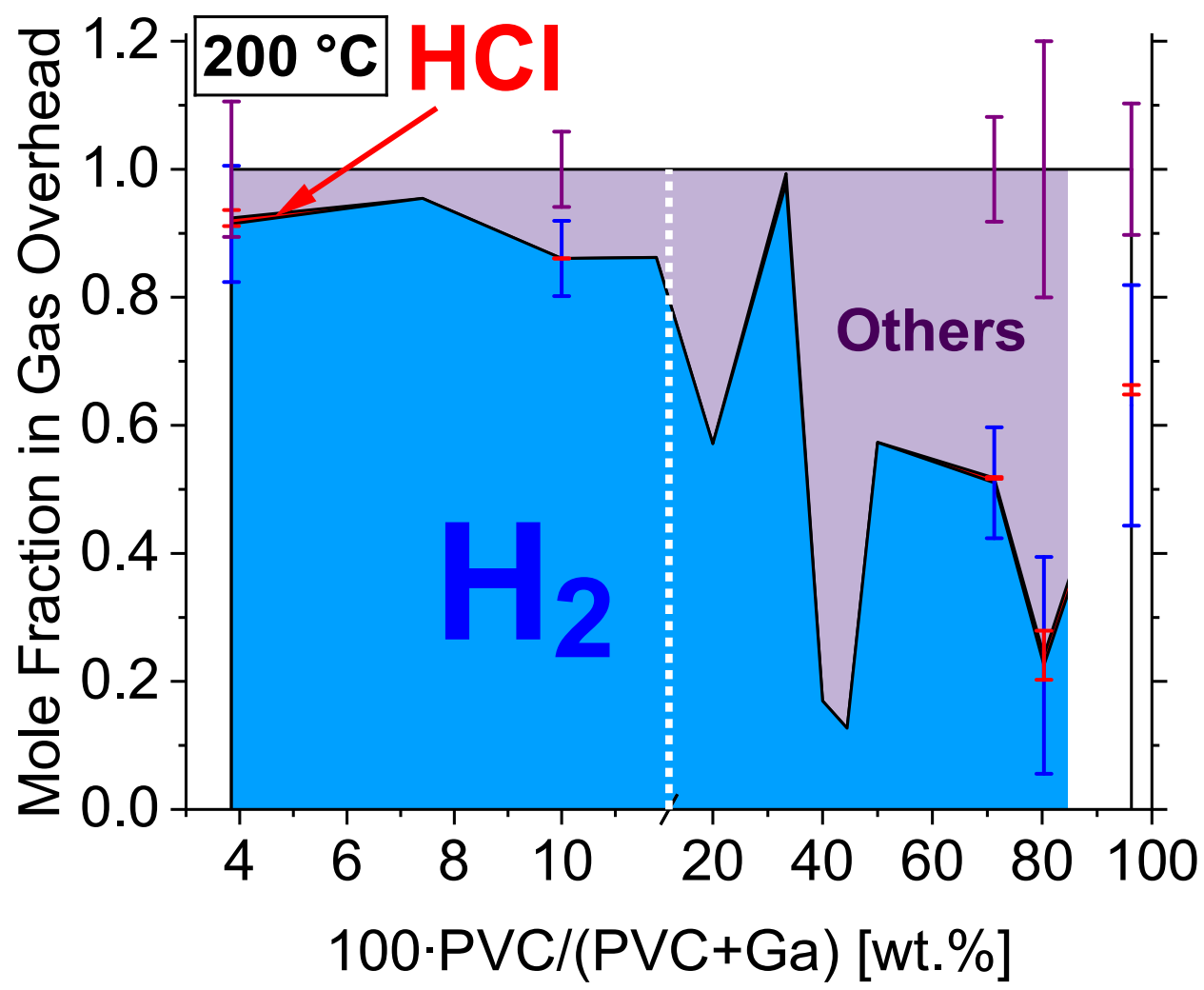

Figure S5. Minimizing the use of Ga LM reduces H_2 selectivity. Further, significant uncertainties are observed. *Reaction conditions: Ga LM catalyst, 0.2-0.4 g PVC, batch mode, Ar atmosphere, 200 °C, 1 h.*

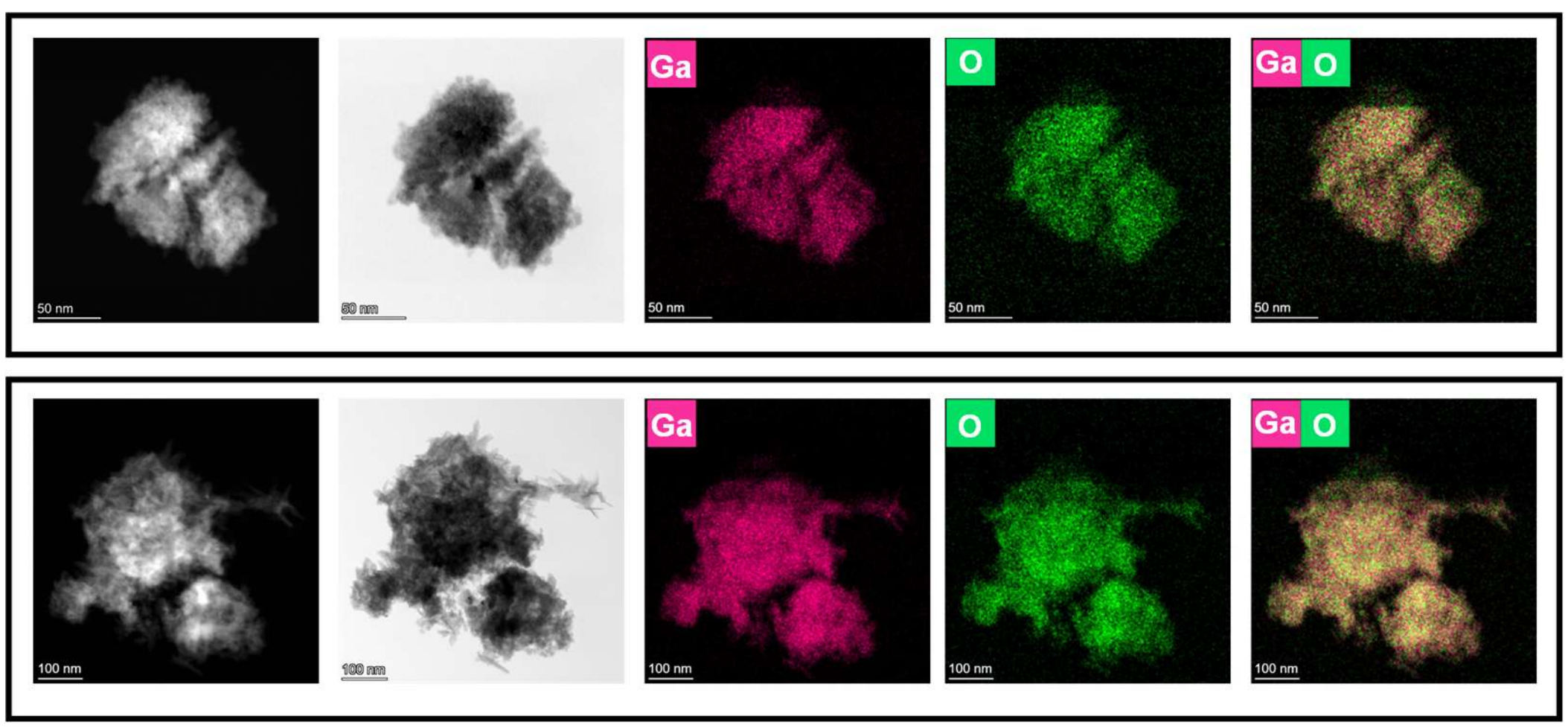

Figure S6. Solid carbonaceous product after PVC conversion using Ga nitrate at 200 °C. STEM annular dark field, brightfield and EDS mapping. Mapping of two representative particles is presented.

To study the ability of metallic Ga to facilitate C–Cl and C–H bond scission, ab initio molecular dynamics (AIMD) simulations were performed. We first fully relaxed the PVC/Ga structure and found no C–Cl or C–H bond scission from geometry optimization (**Figure S7a** left). We then heated up the structure and performed NVT simulations at 900 K for 50 ps. The AIMD simulations revealed significant dechlorination of the PVC chain over Ga LM (**Figure S7**). 10 of 12 chlorine atoms in the simulation cell detached from the polymer backbone in the 50-ps timeframe (**Figure S7b**, **Figure S8**). The process indicated that Ga weakens the C–Cl bonds, thereby promoting chlorine release and the subsequent formation of Ga–Cl bonds. Moreover, we observed three events of C–H bond breaking (**Figure S7b,c**, **Figure S8**): two followed by H transfer (H-1 and H-32) to Ga LM at 17.01 and 32.35 ps; one followed by H transfer (H-14) to a neighbor C atom within the same PVC chain at 47.99 ps. Overall, these findings indicate that metallic Ga can facilitate both dechlorination and dehydrogenation, thus supporting our experimental observations.

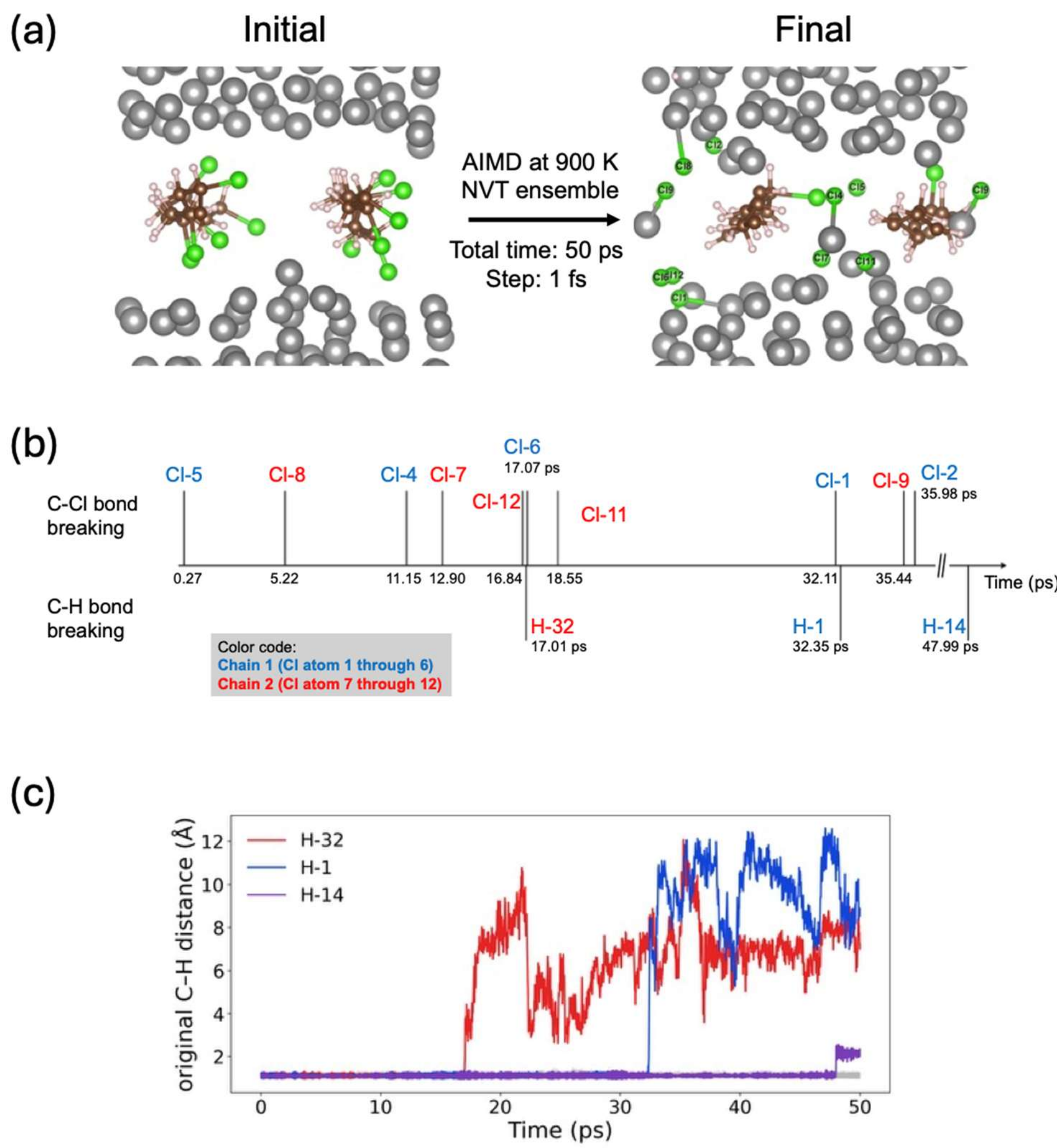

Figure S7. AIMD simulation of PVC/Ga-LM at 900 K for 50 ps: **a)** initial and final structures; **b)** observations of C–Cl and C–H bond breaking events with time; **c)** tracking of C–H distances for the three C–H bond breaking events (note that H-32 and H-1 transfer to Ga LM while H-14 to a neighboring C on the same chain).

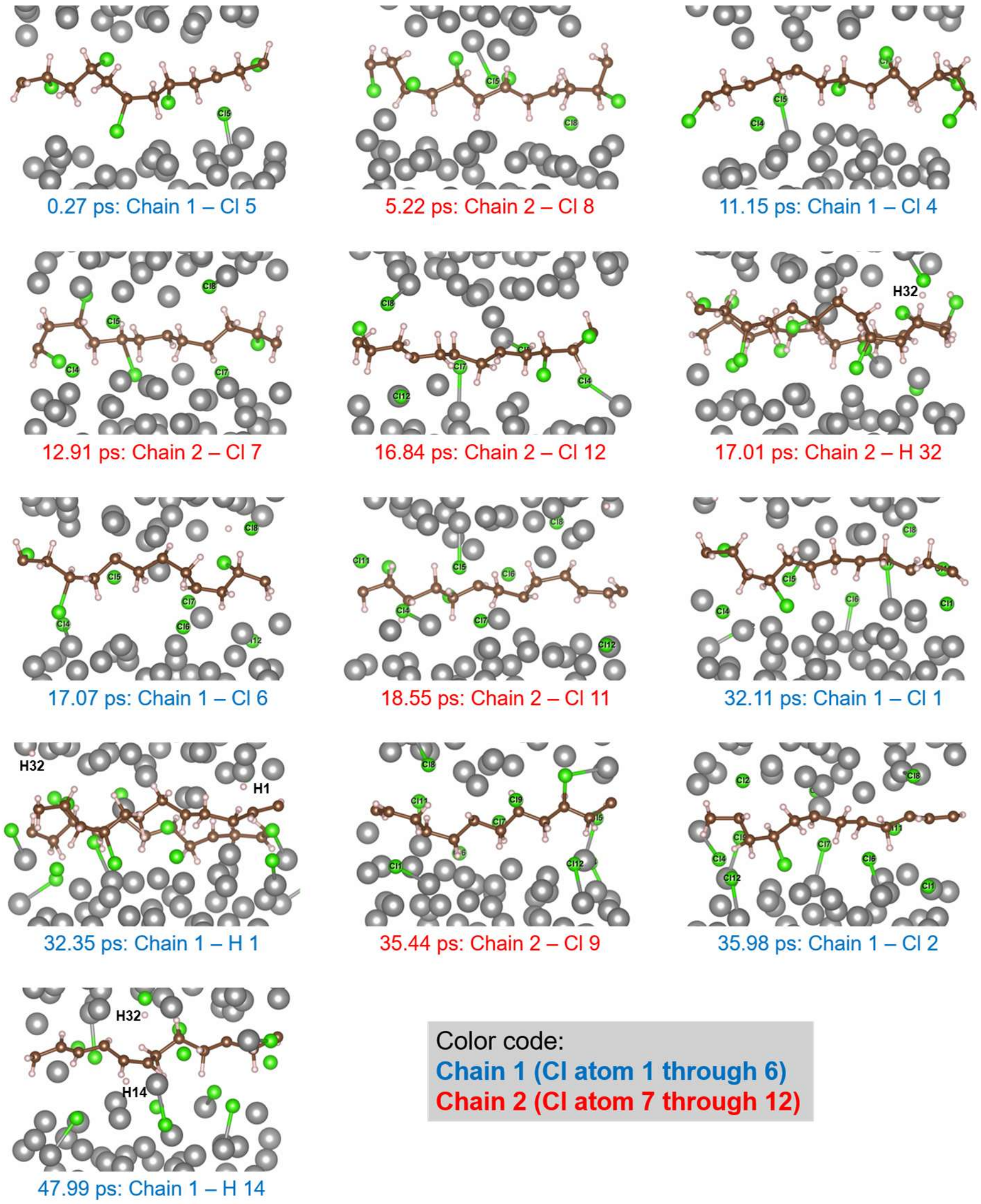

Figure S8. Snapshots of the 50-ps AIMD simulation of PVC/Ga-LM at 900 K at the time points of ten dechlorination events and three C–H bond breaking events.

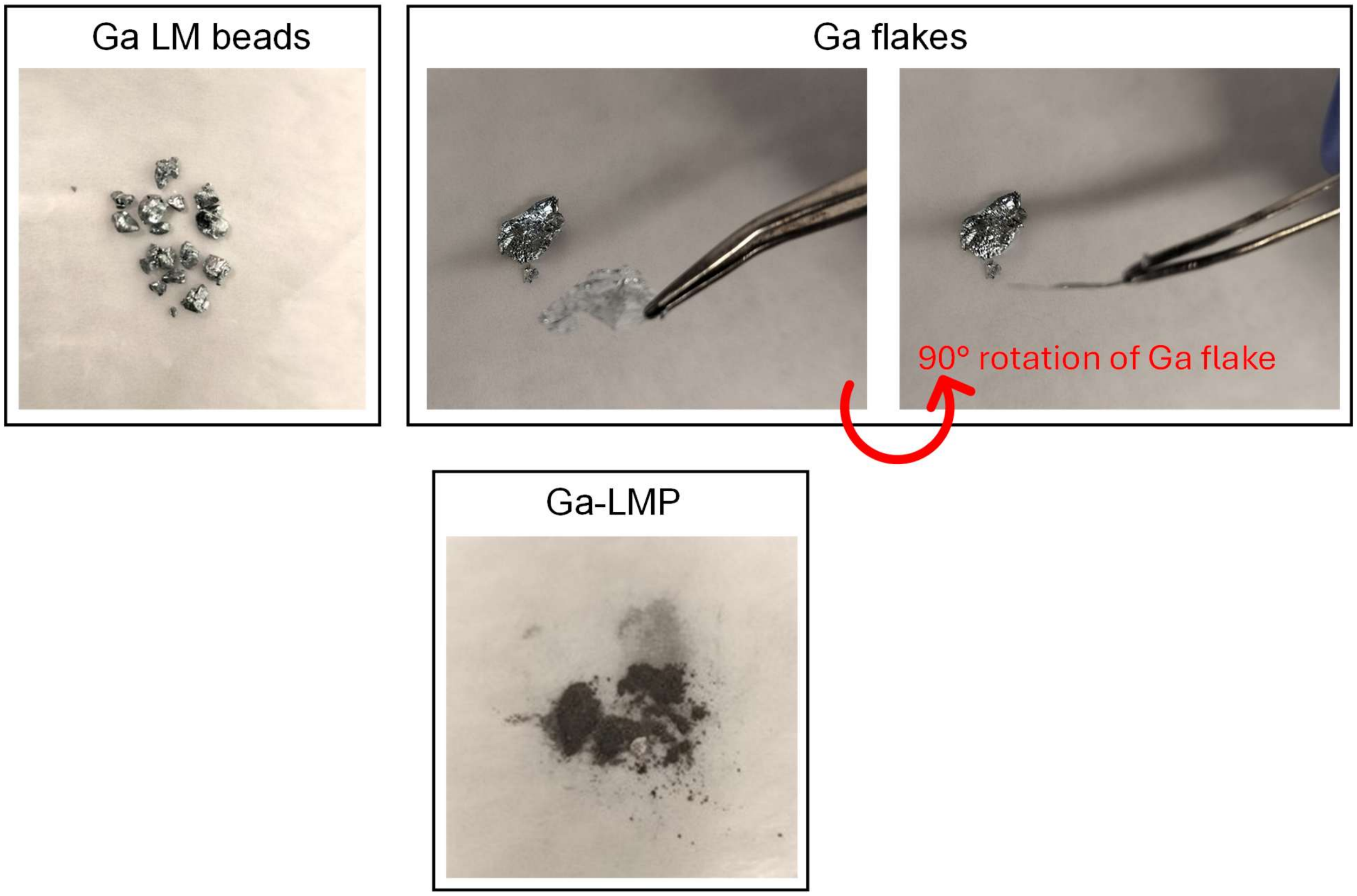

Figure S9. Photographs of Ga beads, Ga flakes, and Ga-LMP-1.5h.

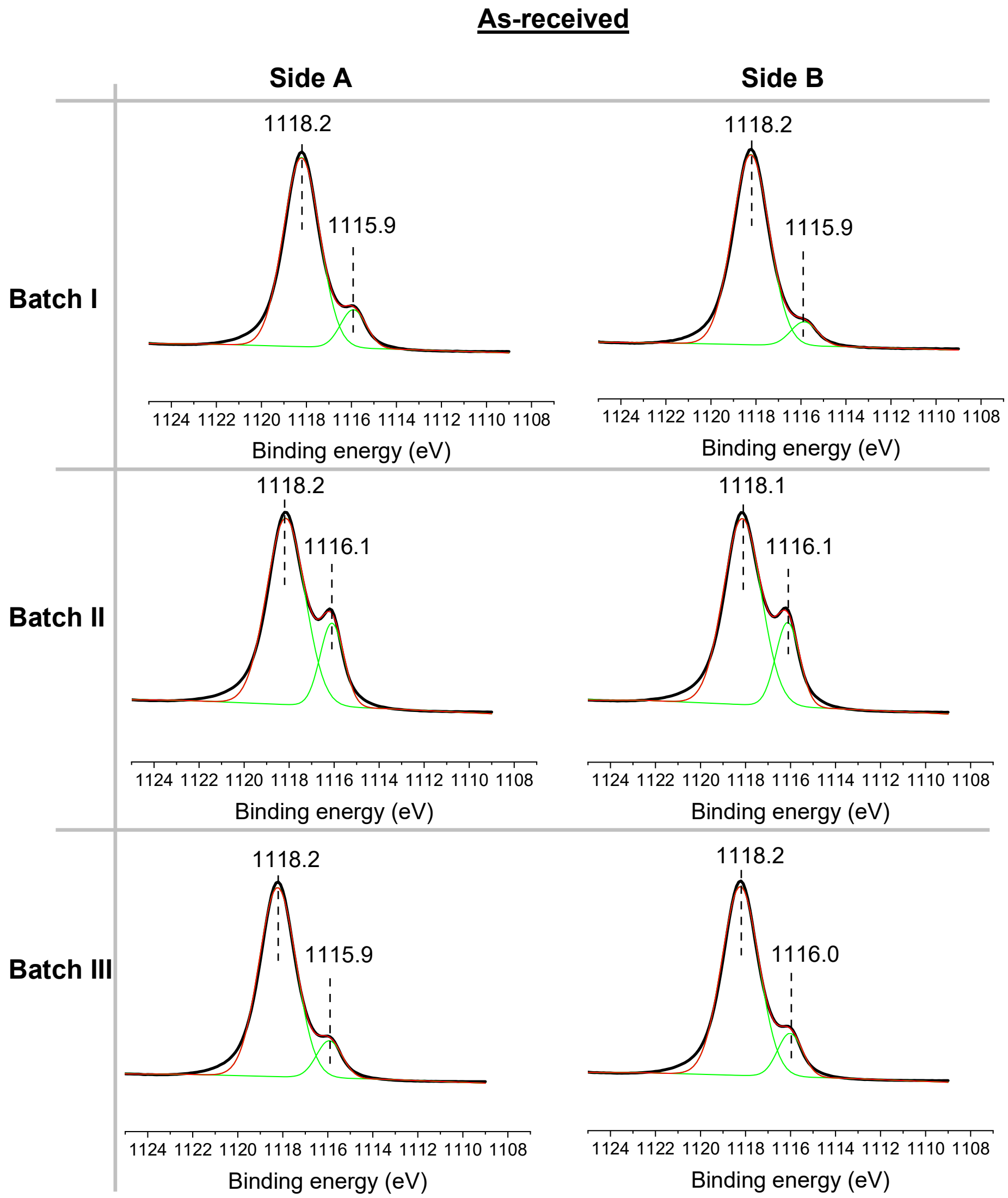

Figure S10. XPS Ga 2p3/2 spectra of as-received Ga-flake samples.

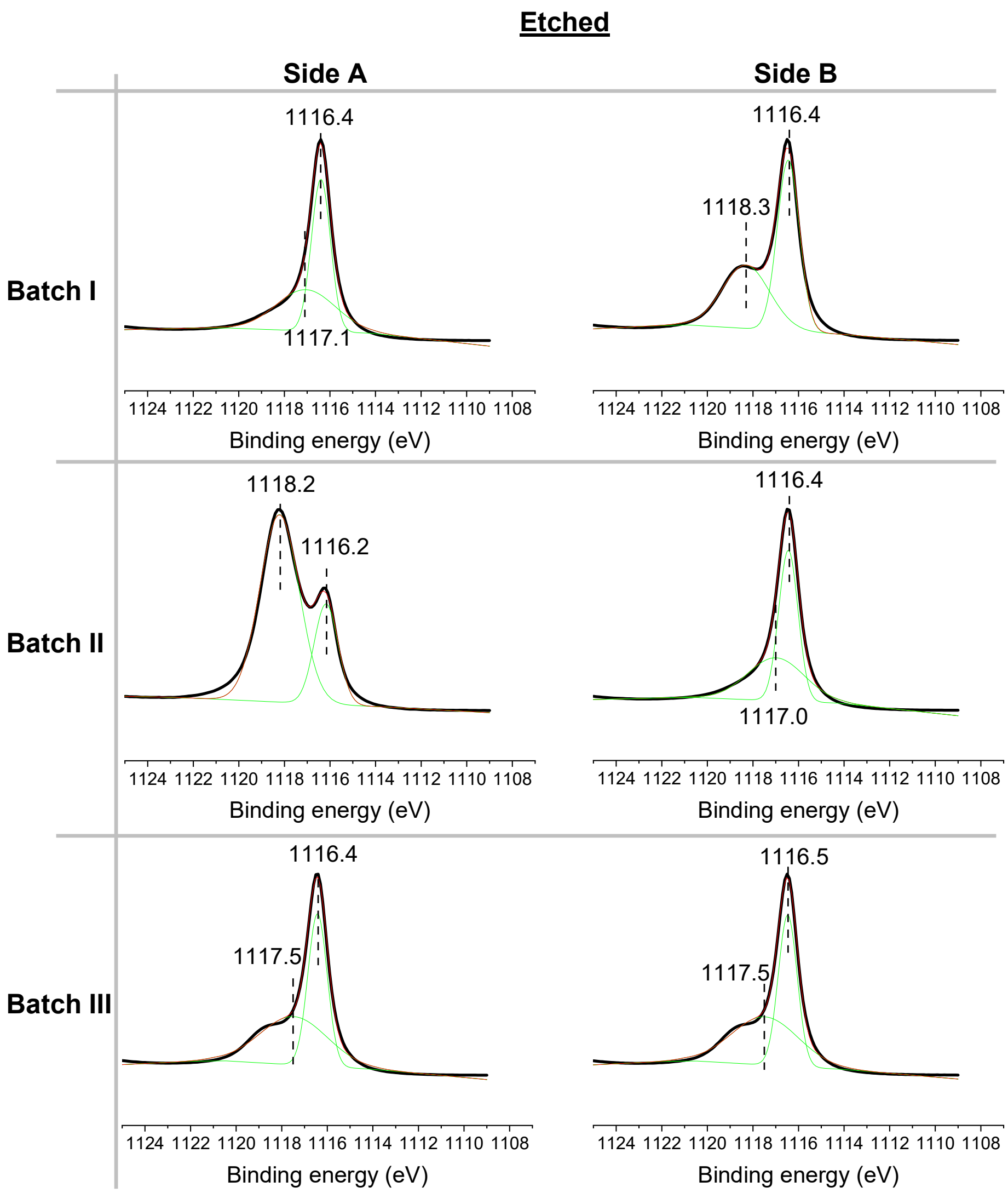

Figure S11. XPS Ga 2p3/2 spectra of etched Ga-flake samples.

Table S1. Ga 2p3/2 XPS fitting for three batches of Ga flakes. Both sides of the flakes were analyzed (side A and side B).

As-received (surface analysis)

	**Side A**			**Side B**			**Average**		
	Ga^{0}	$Ga^{+\delta}$	Ga^{+3}	Ga^{0}	$Ga^{+\delta}$	Ga^{+3}	Ga^{0}	$Ga^{+\delta}$	Ga^{+3}
**Batch I**	12.5	0	87.5	8.4	0	91.6	10.4	0	89.6
**Batch II**	21.7	0	78.3	22.2	0	77.8	21.9	0	78.1
**Batch III**	11.9	0	88.1	13.3	0	86.7	12.6	0	87.4

Etched (sub-surface analysis)

	**Side A**			**Side B**			**Average**		
	Ga^{0}	$Ga^{+\delta}$	Ga^{+3}	Ga^{0}	$Ga^{+\delta}$	Ga^{+3}	Ga^{0}	$Ga^{+\delta}$	Ga^{+3}
**Batch I**	54.1	45.9	0	55.7	0	44.3	54.9	22.9	22.1
**Batch II**	24.5	0	75.5	54.3	45.7	0	39.4	22.9	37.7
**Batch III**	47.5	52.5	0	47.0	53.0	0	47.2	52.8	0

Binding energies: 1115.9-1116.5 eV (Ga^{0}), 1117.0-1117.5 eV ($Ga^{+\delta}$), 1118.1-1118.3 eV (Ga^{+3})

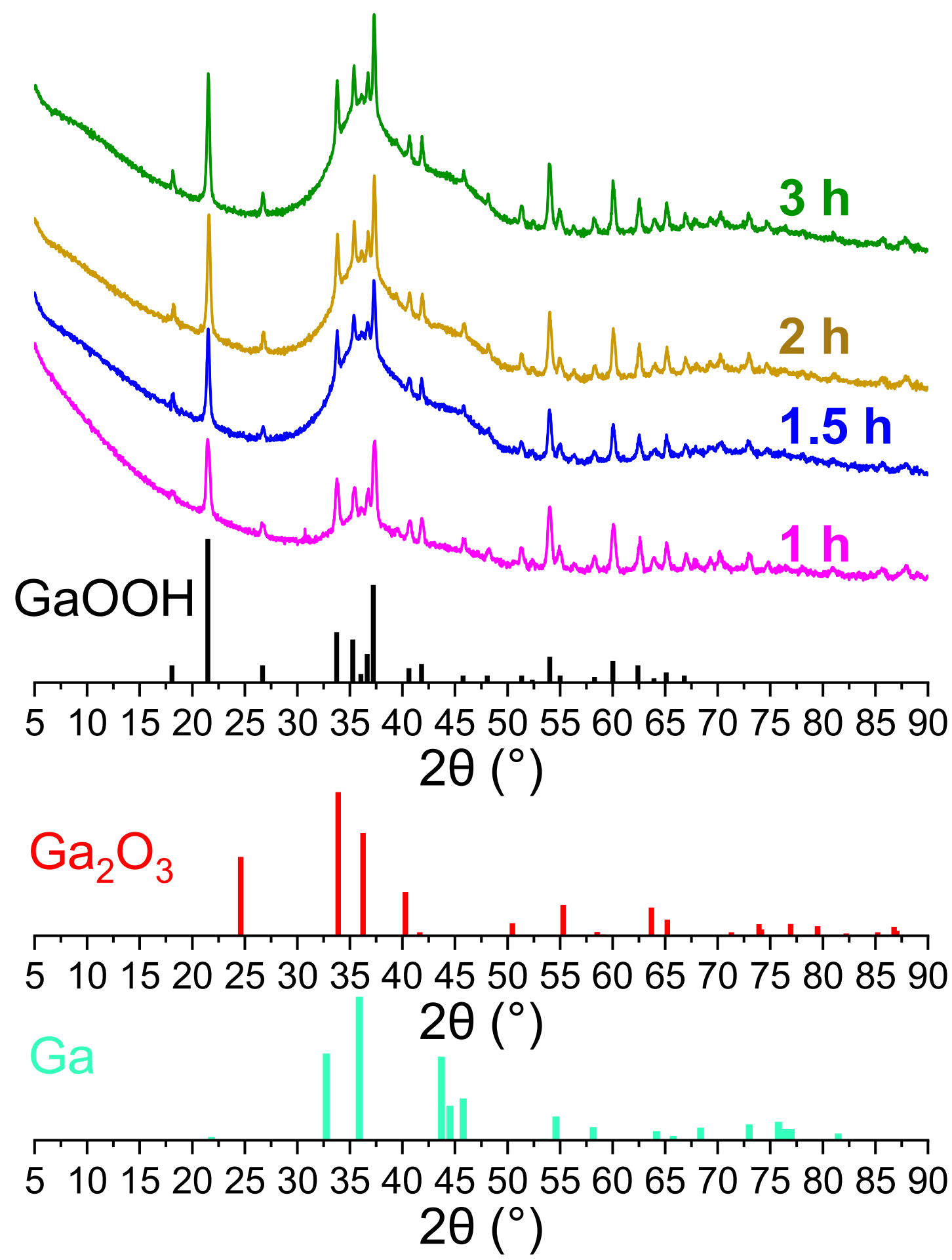

Figure S12. XRD spectra of Ga-LMP samples synthesized with sonication time of 1, 1.5, 2 and 3 h. GaOOH, Ga_2O_3 and Ga references are included.

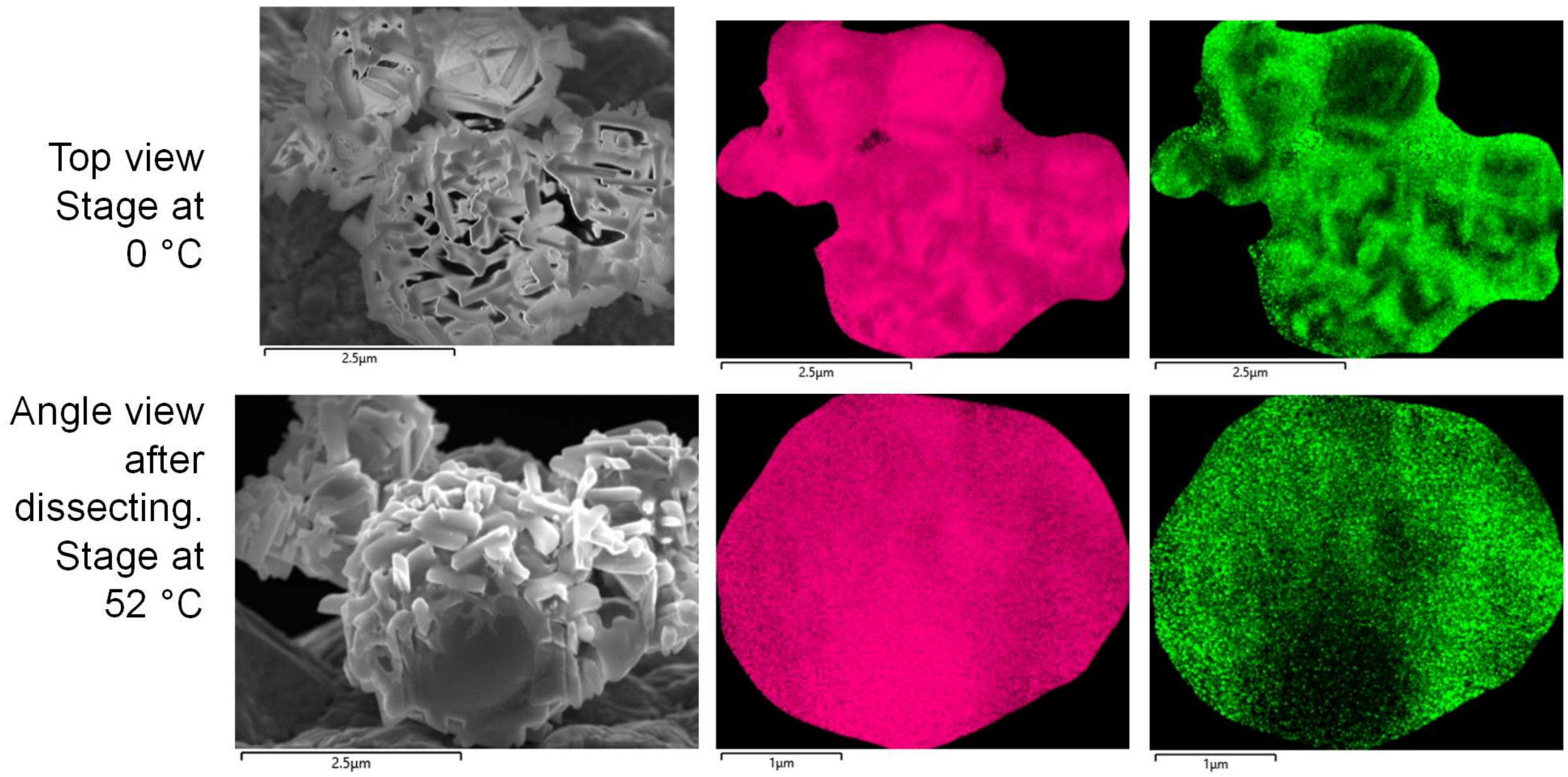

Figure S13. PFIB-SEM-EDS of Ga-LMP-3h.

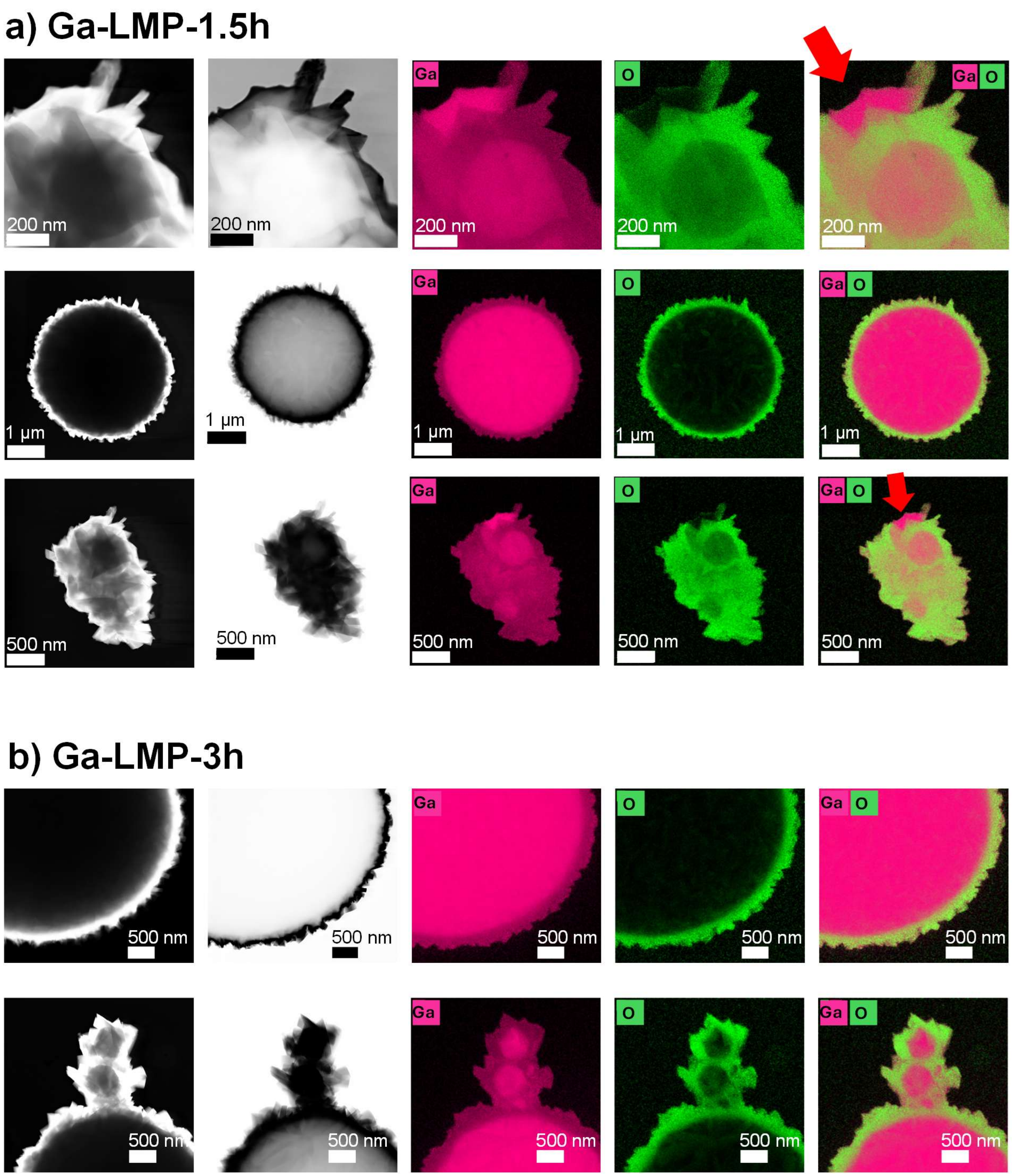

Figure S14. STEM annular dark field, brightfield and EDS mapping of **a)** Ga-LMP-1.5h (red arrow shows an area where the Ga core was partially exposed) and **b)** Ga-LMP-3h.

a)

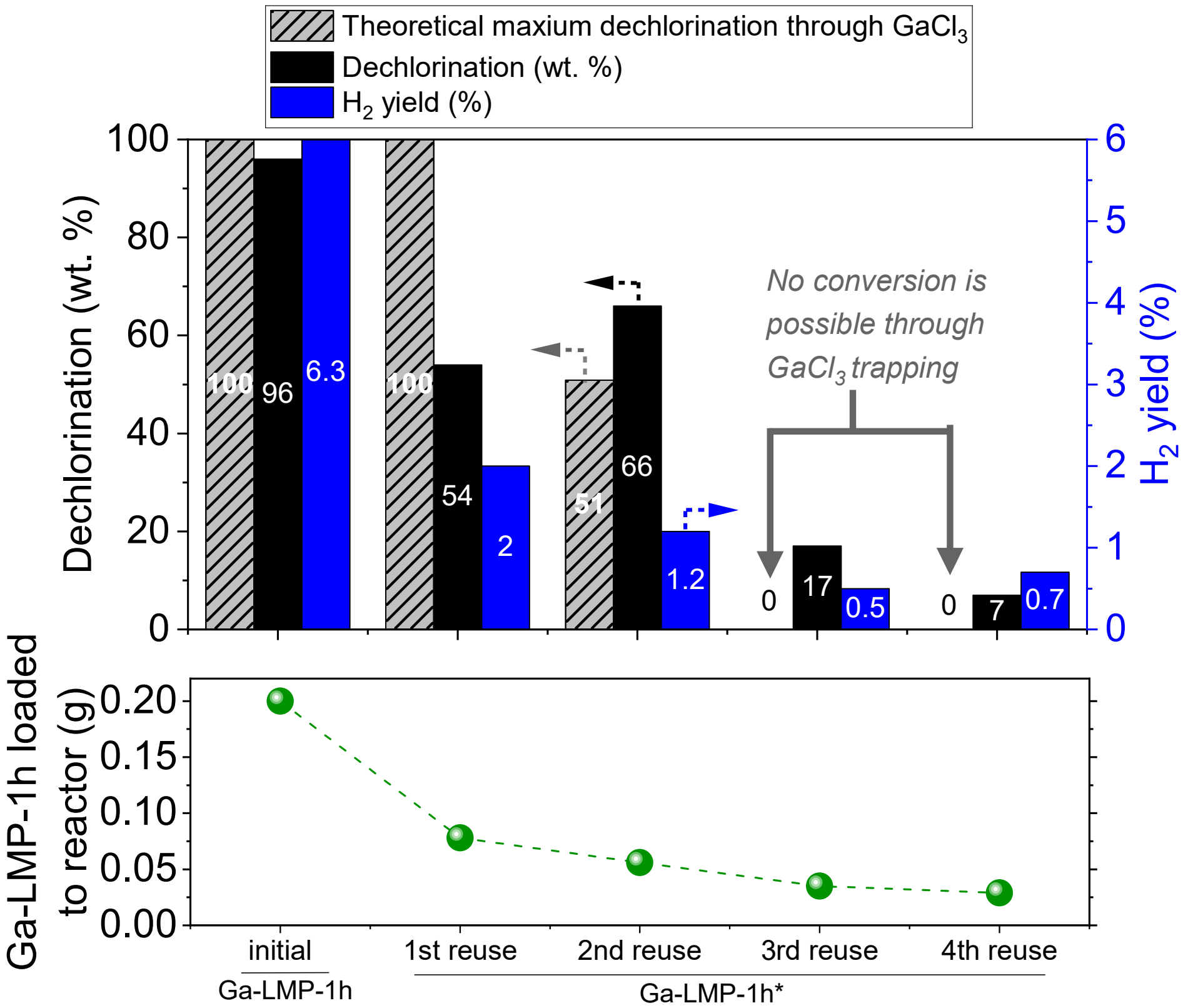

b)

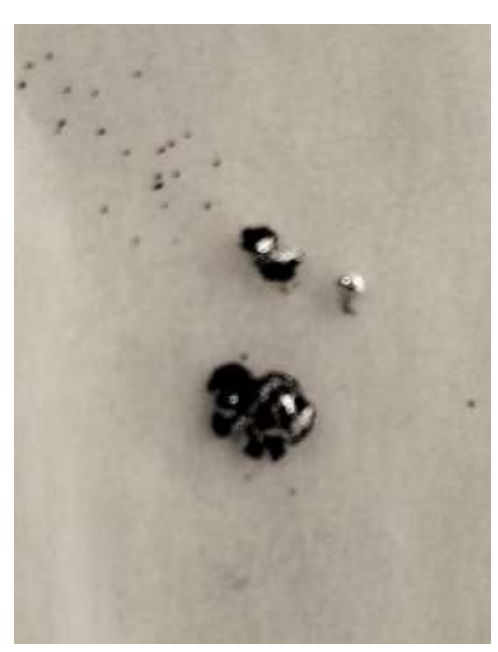

Figure S15. The Ga-LMP-1h catalyst was regenerated into Ga-LM-1h* and reused four times. a) Reusability test for Ga-LMP-1h*. The catalyst was recovered and regenerated through sonication after each 1-h reaction. *Reaction conditions: 1 h, 160 °C, 0.2 g PVC, 0.2 g (initial amount) Ga-LM-1h.* **b)** Photograph of Ga LM bead formed after Ga-LMP-1h has been used to convert PVC. Black powder is dechlorinated PVC that can be removed from the Ga bead with a light tap or shake.

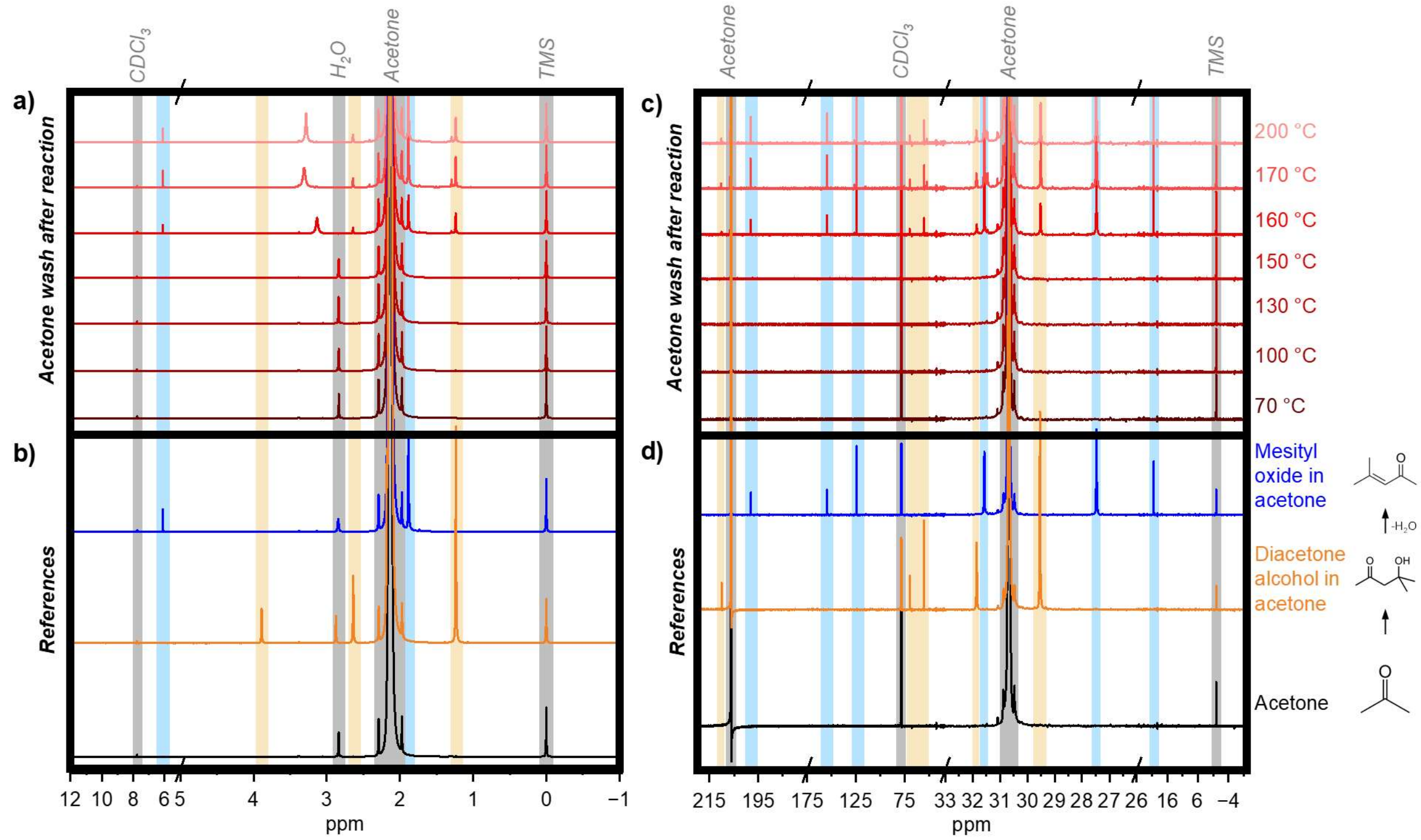

Figure S16. Liquid ^{1}H (**a,b**) and ^{13}C NMR (**c,d**) of: **a,c)** Acetone washes collected after reaction at different temperatures. *Reaction conditions: 1 h, 0.2 g PVC, 0.049 g Ga-LMP-1h*. **b,d)** Mesityl oxide, diacetone alcohol and acetone references. All solutions analyzed were mixed with equal volume of $CDCl_3$ before NMR characterization.

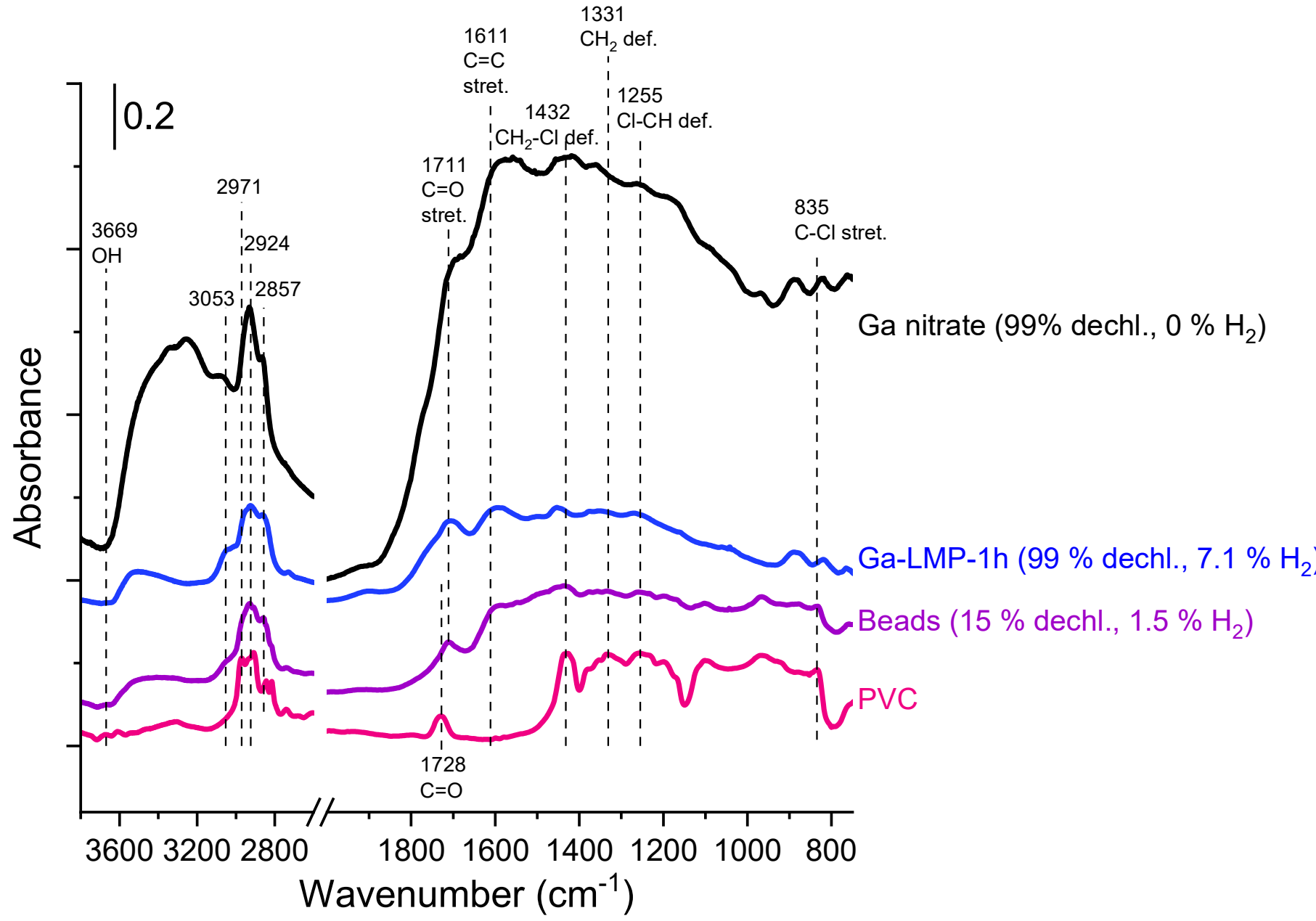

Figure S17. Ex situ IR spectroscopy of solid products obtained at 200 °C using Ga nitrate, Ga beads and Ga-LM-1h. Spectrum of fresh PVC is included as reference. *Reaction conditions: 1 h, 1200 RPM, 200 °C, 0.2 g PVC, 0.049 g Ga LMP (or Ga LM beads). Background: 25 °C, Ar atmosphere, KBr.*

The solid product obtained using Ga beads presented C–Cl features (835 cm^{-1}) since only 15 wt. % dechlorination was attained. Ga beads produced some H_2 (1.5 % yield) and the solid product obtained presented a shoulder at 3053 cm^{-1} corresponding to C-H stretching in alkenes, and a feature at 1611 cm^{-1} corresponding to C=C bonds. The solid product obtained using Ga-LMP-1h (99 wt. % dechlorination, 7.1% H_2 yield) presented more defined bands at 3053 and 1611 cm^{-1}, corresponding to the creation of more C=C bonds. The C–Cl feature at 835 cm^{-1} was less evident. The solid product obtained using Ga nitrate –where no H_2 was produced– did not present a band at 3053 or 1611 cm^{-1}, showing that H_2 yield originates from generation of C=C bonds.

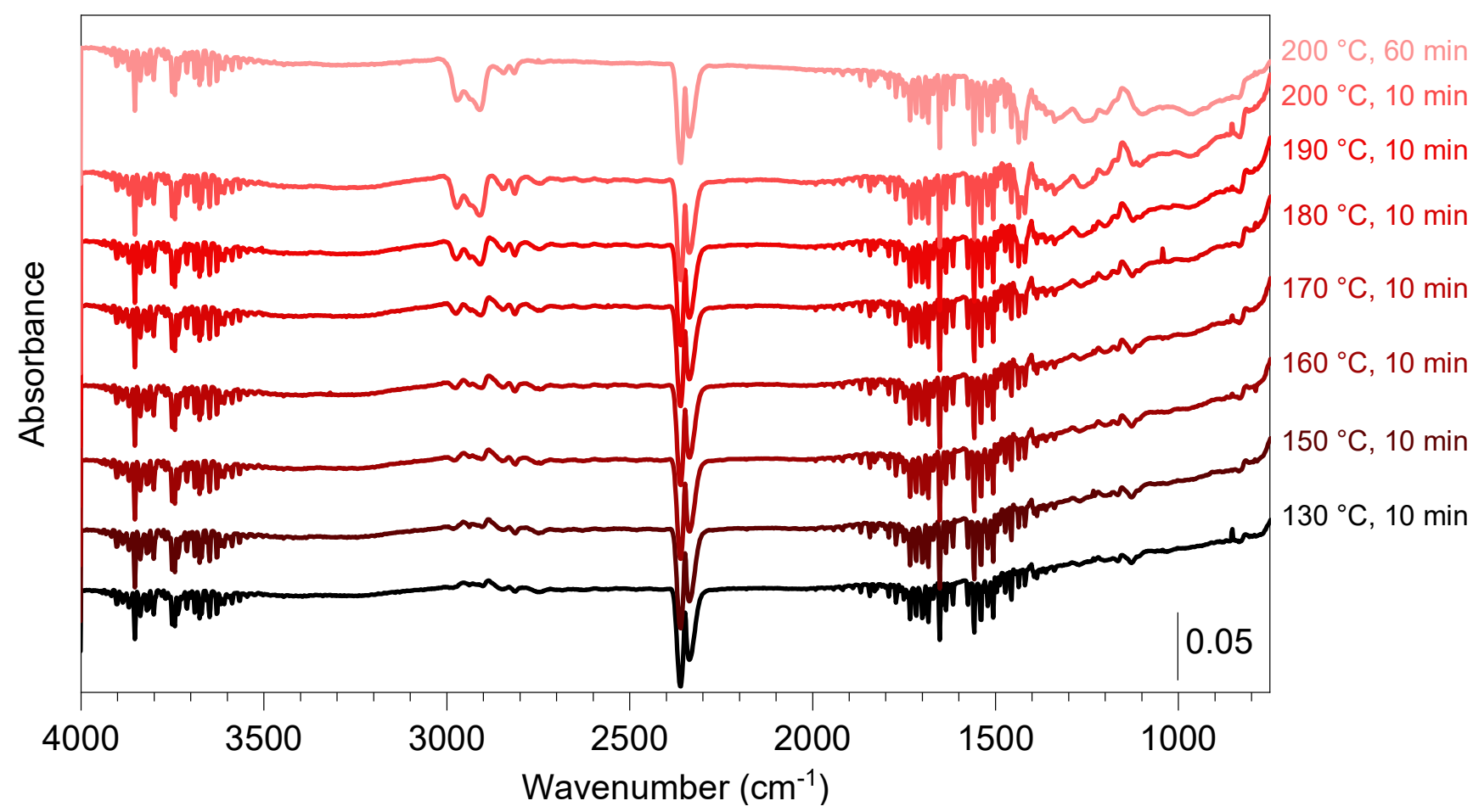

Figure S18. In situ DRIFTS using a 1:1 (wt./wt.) mixture of PVC:Ga-LMP-1h. Temperature was increased stepwise and held at for 10 min at 130, 150, 160, 170, 180 C and at 200 C for 1 h. Extended wavenumber range.

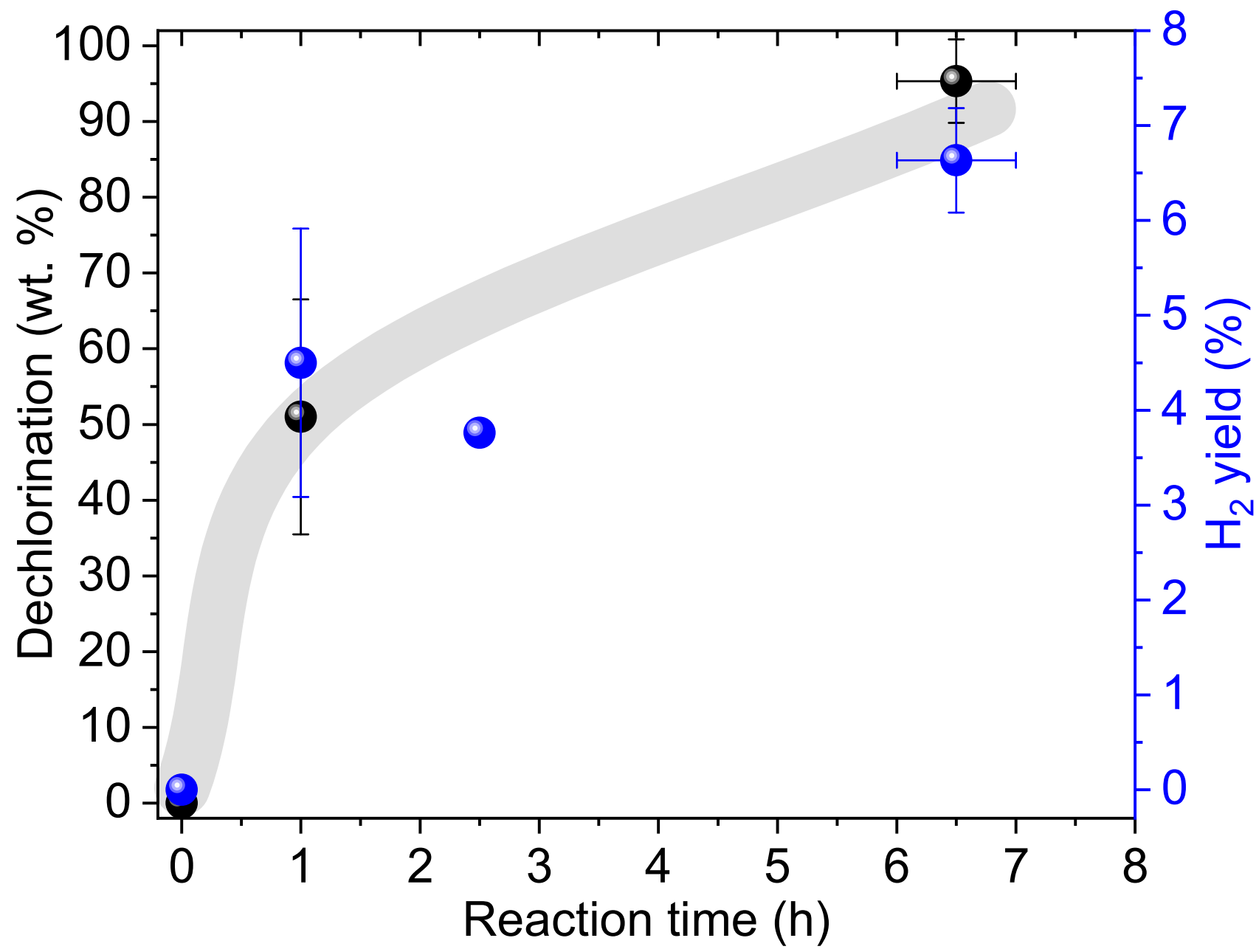

Figure S19. PVC dechlorination and H_2 yield as a function of reaction time using Ga-LMP-1h. *Reaction conditions: 160 °C, 0.2 g PVC, 0.049 g Ga-LMP-1h.*
